# Significance of Nano Transition Metal Complexes as Anticancer and Antibacterial Therapeutic Agents

**DOI:** 10.3390/ijms262110516

**Published:** 2025-10-29

**Authors:** Abdulaziz Alshehab, Ameena Haider, Laila Jaragh-Alhadad

**Affiliations:** 1 College of Graduate Studies, Kuwait University, Kuwait City 13060, Kuwait; 2 Department of Chemistry, Faculty of Science, Kuwait University, Kuwait City 13060, Kuwait; 3 Department of Biology, Faculty of Science, Kuwait University, Kuwait City 13060, Kuwait; ameena.haider@ku.edu.kw

**Keywords:** transition metal complexes, synthesis, anticancer effect, DFT calculations, antibacterial activity

## Abstract

Metal complexes have been utilized in medicine for thousands of years in various ways, with positive benefits. They are well recognized as delivery and diagnostic agents with anti-infective, antimicrobial, antidiabetic, and neurological properties. Based on this, Co, Zn, and Cu complexes were synthesized using a Schiff base reaction and were fully characterized. The complexes were then assessed using breast cancer cell line (MDA-MB-453), and the data show that the Cu–ligand complex was the most potent anticancer agent, followed by the Zn complex, and then the Co complex. This result is supported by DFT theoretical calculations. In addition, the complexes were tested for their biological activities against both Gram-positive and Gram-negative bacteria, and the data reveal that Cu had a strong inhibitory effect on the growth of both types of bacteria. In summary, the copper complex proved to be a potent anticancer and antibacterial agent, and it can be considered for utilization in future therapies.

## 1. Introduction

The field of medicinal chemistry holds metals in high regard. Groups three through twelve on the periodic table are known as the “d block” elements, or transition metals [1]. Filling up their d shells led to the development of coordination complexes. A metal complex, also known as a coordination compound, is a structure made up of a central metal atom bound to a variety of nearby ligands or anions [1,2]. These complexes have the capacity to interact and react with biomolecules in novel ways and through different modes of action. Metal ions have significant effects on biological processes, and the study of how transition metal chemistry might be used to treat or diagnose illnesses is an attractive field in medicinal chemistry [3], which began in the past few decades with platinum complexes, such as the cancer drug cisplatin [1]. There is a global epidemic of diseases brought on by bacterial, fungal, and viral infections [4]. Other significant health issues include diabetes [5], cancer [6], and malaria [6,7]. Various metals and nonmetals in the periodic table have demonstrated their significance in the healthy functioning of the human body [8,9]. Specifically, d-block transition metal elements, such as manganese (Mn), iron (Fe), cobalt (Co), copper (Cu), zinc (Zn), and molybdenum (Mo), are essential for regular biological functioning and homeostasis [8,10]. Either a deficiency or an excess of transition metals can lead to various diseases in the human body [9], including Alzheimer’s disease [11], Parkinson’s disease [12], cardiovascular diseases [13], cancer [14], and diabetes [8,15]. Therefore, it becomes necessary to discover new agents that may be employed as therapeutic medications for bacterial and cancer treatments.

Schiff base reaction is a kind of condensation reaction where a primary amine and a carbonyl molecule (an aldehyde or ketone) combine. The amine is added nucleophilically to the carbonyl group in the reaction, and then a water molecule is removed in a dehydration step. The water generated by these reversible processes is frequently eliminated by employing techniques like molecular sieves or azeotropic distillation. Schiff bases are selective toward metal ions and form complexes by transferring electrons from the active ends they contain to the metal [16,17,18,19].

Based on this information, researchers have synthesized numerous transition metal complexes for disease treatment and diagnosis, including those consisting of cobalt, copper, and zinc [20,21,22]. First, cobalt is a naturally occurring element that forms the coenzyme vitamin B-12 and exhibits distorted octahedral geometries, forming stable complexes with many ligands, including water, chloride ions, and ammonia [23,24]. Studies have shown that cobalt complexes can be utilized as antiviral and antibacterial agents [25], anticancer agents [26], and antimalarial agents [8,27]. Second, copper is essential for several vital biological activities [8], such as photosynthesis, iron metabolism, mitochondrial respiration, catalytic cofactor, scavenging of free radicals, the transmission of electrons inside biological molecules, certain neurological processes [28,29], and the redox chemistry of enzymes, all of which are essential to cell physiology and can happen because of the two different redox states of reduced copper (I) and oxidized Cu (II) [30]. Studies have also shown that copper complexes exhibit anticancer activities [31,32] and function as antibacterial [32] and antimalarial agents [33]. Third, zinc is a catalytic metal that plays a crucial role in biological signaling systems [34], protein metabolism, and the immune system [35]. Zinc is a structural metal that occurs in a variety of RNA- and DNA-binding proteins known as zinc fingers [36]. Additionally, research studies have demonstrated that zinc complexes exhibit anticancer and antibacterial properties [37,38].

**Figure 1:**
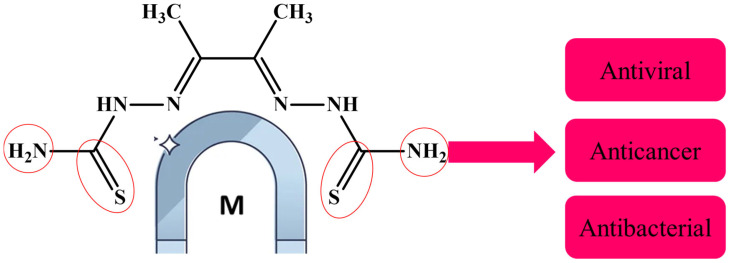
Thiosemicarbazone is a metal chelator because of its carbon–nitrogen and carbon–sulfur bonds.

Several ligands have played a significant role in the development of coordination chemistry and form stable complexes with many transition metals [16,17,18]. In this study, a thiosemicarbazone was used to form Co, Cu, and Zn complexes. Thiosemicarbazones’ inherent biological activity, structural flexibility, and multifunctional metal-chelating capabilities have attracted a lot of attention [39]. Before their anti-tumor effect was discovered, thiosemicarbazones were known to be metal chelators. They have a versatile thiourea structure that allows them to incorporate other substituents or functional groups in addition to the C=N and C=S bonds in their own structure, which is advantageous for metal coordination [39,40], and they exhibit a wide variety of biological activity, such as antiviral [4], antifungal [17,25], and anticancer properties [40] (Figure 1).

**Figure 2:**
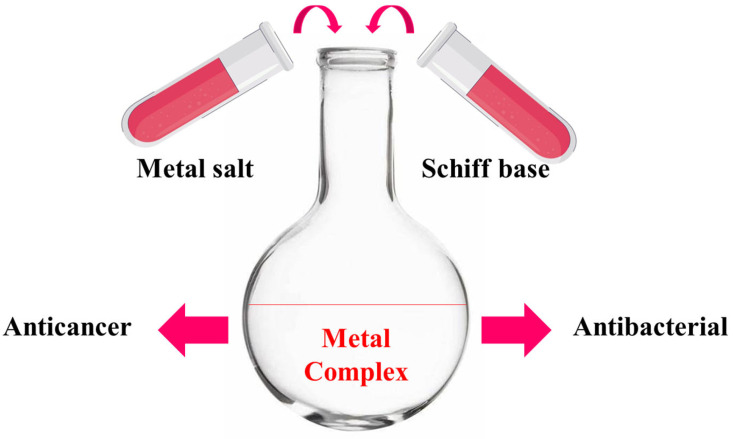
Schematic representation, starting from synthesis to biological evaluation.

A study on thiosemicarbazone metal complexes is novel when we use a different metal, a new synthetic process, or a new ligand structure; when we focus on a biological activity that has not been thoroughly investigated; or when a combination of powerful computational and experimental techniques is employed. Studies that focus on creating a next-generation molecule to boost a lead drug’s efficacy against germs or cancer, for instance, are considered unique [41,42]. Therefore, cobalt(II), copper(II), and zinc(II) metal complexes have been synthesized and characterized by CHNS analysis, mass spectroscopy, proton and carbon NMR, FTIR, thermal gravimetric analysis, molar conductivity, differential scanning calorimetry, scanning electron microscopy, and dynamic light scattering. The metal complexes were biologically evaluated for their anticancer activity using a breast cancer cell line, and this was supported by a molecular geometry and computational methodology study. Additionally, the antibacterial activity of the metal complexes was evaluated using both Gram-positive and Gram-negative bacteria, as shown in Figure 2.

## 2. Results

A Schiff base reaction was used to synthesize the ligand thiosemicarbazone, which was then used to form metal complexes at the nanoscale. The obtained ligand and its metal complexes are air stable. The complexes exhibit various colors. A solubility test indicates that these complexes are insoluble in water but soluble in polar aprotic solvents, such as DMSO and DMF. The molar conductivities (Λ^a^) of the Co, Cu, and Zn complex results were measured in a 10^−3^ M DMSO solution. The low conductivity values (less than 50 µS/cm) are 5.83, 4.34, and 21.52 (Ω^−1^ cm^2^ mol^−1^), respectively, indicating their non-electrolytic nature. This confirms that chloride ions are not present outside the coordination sphere. The Co, Cu, and Zn complexes were created and synthesized, and their structures were fully characterized. The complex formulas, physical characteristics, elemental analyses, thermal properties, molar conductivity, magnetic moment, and formula weights (LCMS) derived from mass spectra are listed together in Table 1. The data illustrate that Co^2+^ and Cu^2+^ complexes were formed in a 1:1 molar ratio (1 L:1 M) with the formula [M(L)].2H_2_O, where M = Co^2+^ or Cu^2+^, while Zn^2+^ acts as (2 L:1 M) with formula [Zn(H_2_L)_2_].

### 2.1. Elemental Analysis, Electronic Spectra, and Magnetic and Physical Measurements

The elemental analysis is in good agreement with the calculated results for the proposed formula of the metal complexes, as described in Table 1.

### 2.2. Mass Spectra of Metal–Ligand Complexes (MS)

**Figure 3:**
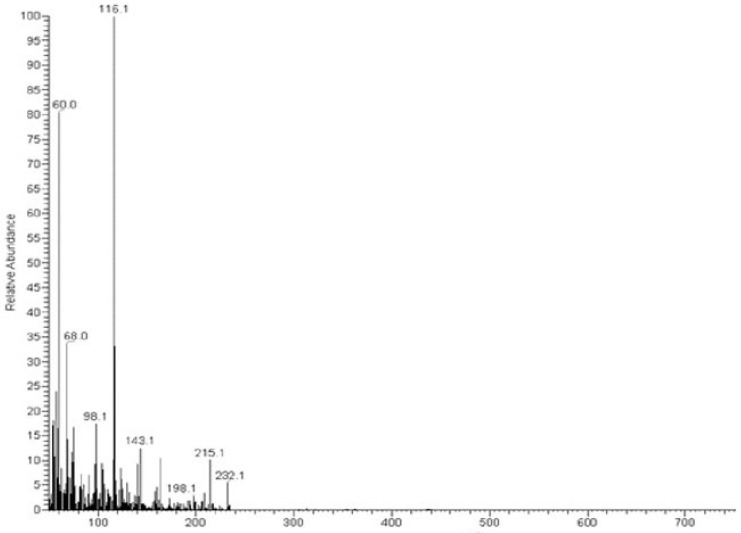
Mass spectrum of ligand thiosemicarbazone (H_2_L).

**Scheme 1:**
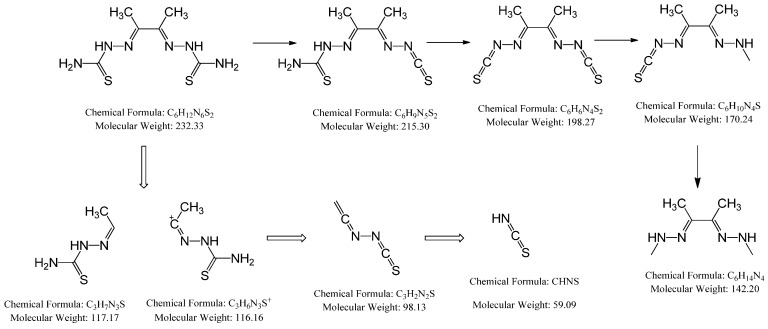
Schematic representation of how the molecular peaks will break.

**Figure 4:**
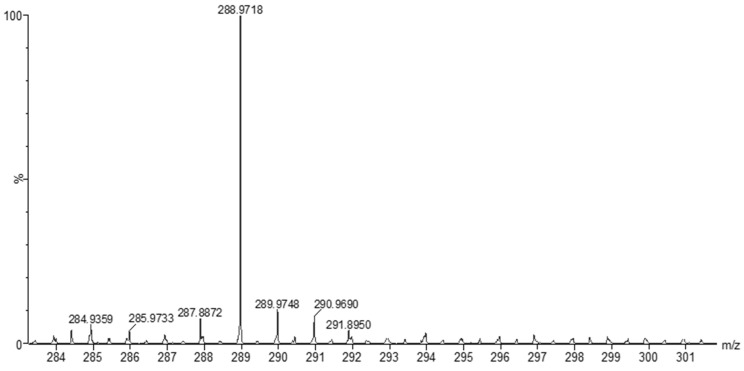
Mass spectrum of [Co(L)].2H_2_O.

**Figure 5:**
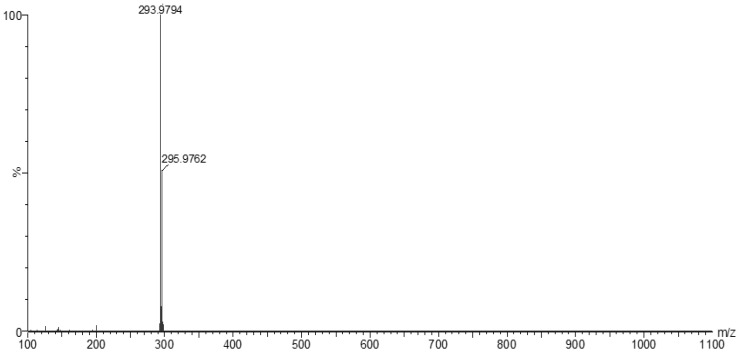
Mass spectrum of [Cu(L)].2H_2_O.

**Figure 6:**
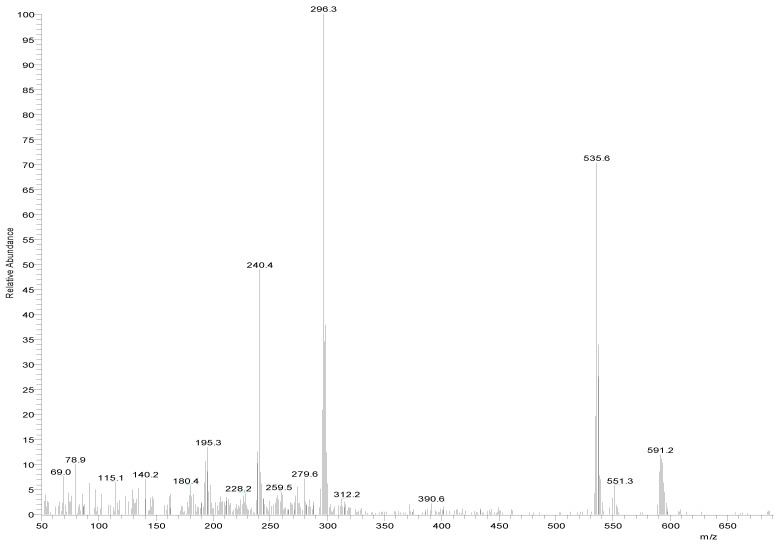
Mass spectrum of [Zn(H_2_L)_2_].

The MS of H_2_L (Figure 3) showed a molecular peak at *m*/*z* = 232.1 (7%), with weak peaks surrounding it due to the presence of ^13^C and ^15^N isotopes. The other positive ions achieve peaks at 215.1 (15%), 174.2 (3%), 116.0 (100%), 86.1 (6%), and 56.07 (32%) mass numbers (Scheme 1). The intensities of these peaks indicate the stability of these fragments. The MS of [Co(L)].2H_2_O displayed a molecular ion peak of 100% intensity at *m*/*z* = 288.97, corresponding to [Co(L)], as shown in Figure 4. The species that might have formed after the removal of 2H_2_O could be observed, with the small peaks at 250, 163, and 158 representing sequential degradation of the molecule. The MS of [Cu(L)].2H_2_O showed a peak of 100% intensity at *m*/*z* = 293.97, corresponding exactly to [Cu(L)]. This species might have formed after the removal of 2H_2_O. The positive ion peak at 235 (2%) might be due to the removal of methyl and amino groups. The other positive ions showed peaks at mass numbers 192 (4%), 178.0 (1%), 164 (65%), 141 (13%), and 124 (16%), representing sequential degradation of the molecule (Figure 5). With regard to the spectrum of [Zn(H_2_L)_2_], a peak appeared at *m*/*z* = 532.06 (calculated: 535.6), representing the molecular ion peak with 75% intensity, and a peak of intensity of 100% appeared at 296.3, corresponding to [Zn(H_2_L)], as shown in Figure 6.

### 2.3. Nuclear Magnetic Resonance (^1^H and ^13^C)

**Figure 7:**
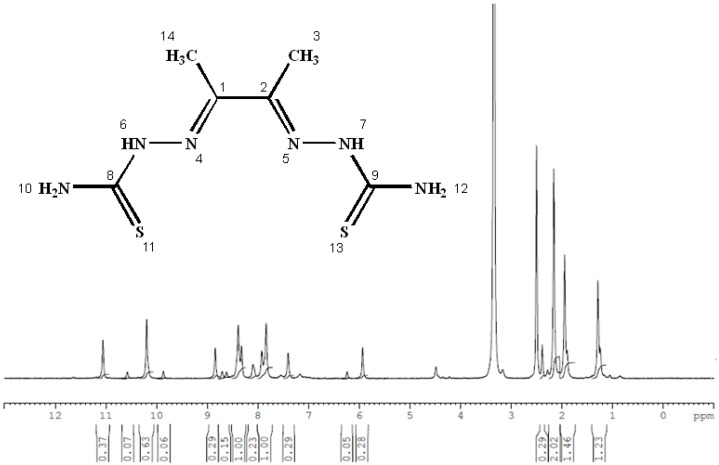
The ^1^H NMR spectrum of the thiosemicarbazone ligand (H_2_L).

**Figure 8:**
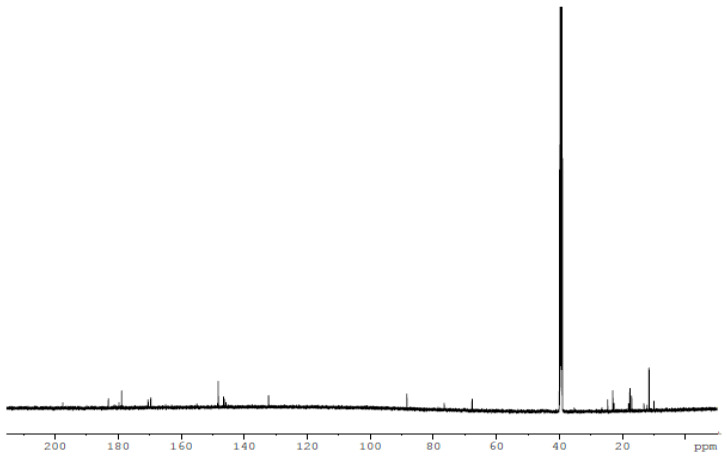
The ^13^C NMR spectrum of the thiosemicarbazone ligand (H_2_L).

**Figure 9:**
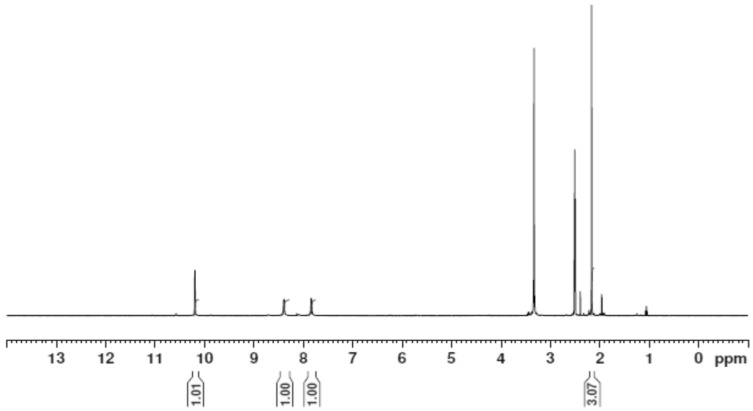
The ^1^H NMR spectrum of the [Zn(H_2_L)_2_] complex.

**Figure 10:**
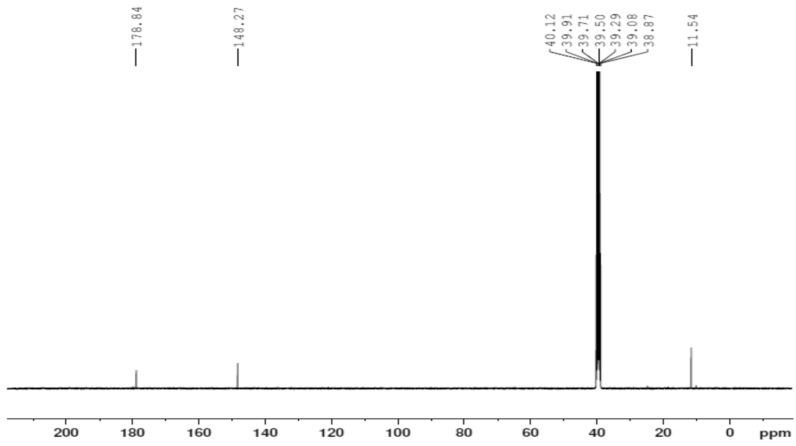
The ^13^C NMR spectrum of the [Zn(H_2_L)_2_] complex.

The ^1^H NMR and ^13^C NMR spectra of the free ligand and Zn–ligand complex were recorded and are summarized in Table 2 and Figure 7, Figure 8, Figure 9 and Figure 10. The ligand ^1^H-NMR spectrum in DMSO-d_6_ agrees with the proposed chemical structure. The two chemical shifts were noted as singlet at 11.061 and 10.197 ppm (s, 1H, NH^6^) and (t, 1H, NH^7^), while the doublet chemical shifts were noted at 8.390 (dd, 2H, ^10^H_2_N) and 8.101(d, 2H, ^12^H_2_N). The spectrum also showed chemical shifts at δ = 1.947 ppm for the CH_3_ group at positions (3 and 14), which were attributed to the methyl group protons. The ^1^H-NMR spectrum confirms that the thiosemicarbazone exists only in the thioketo form; there is no evidence for the existence of the thioenol form. This conclusion was supported by the existence of chemical shifts related to (NH) and the complete lack of a chemical shift related to (SH) of the thioenol form [43]. The ^13^C-NMR spectrum of the ligand thiosemicarbazone (H_2_L) displayed a chemical shift at 183.12 ppm, assigned to the thioketone carbon atom (8,9C=S) [44,45]. The imine carbon (1,2C=N) chemical shift appeared at 148.37 ppm [44,45]. The peaks appearing at 11.55 and 13.81 ppm can be attributed to the methyl group carbon atoms (3,14CH_3_). It is worth noting that we did not run NMR for the [Cu(L)].2H_2_O and [Co(L)].2H_2_O complexes because they are paramagnetic complexes that significantly impact the interpretation of NMR spectra by causing broadening and large chemical shifts. The ^1^H-NMR spectra of the Zn^2+^ complex showed that the thioamide protons appeared at 10.208 ppm, clarifying that the Zn^2+^ is bonded to the thioketo tautomer via the deprotonated thioamide sulfur atom. The ^13^C-NMR spectrum of the Zn^2+^ complex showed that the thioamide (C=S) signal shifted upfield by about 5 ppm compared to the parent ligand, thiosemicarbazone. This upfield shift may be due to the movement of electron density from the thioamide moiety to the Zn^2+^ ions upon chelation, which could have caused the thioamide carbon nuclei to be de-shielded, hence the upfield shift [46].

### 2.4. Infrared (FTIR) Spectra of Co, Cu, and Zn Complexes

**Figure 11:**
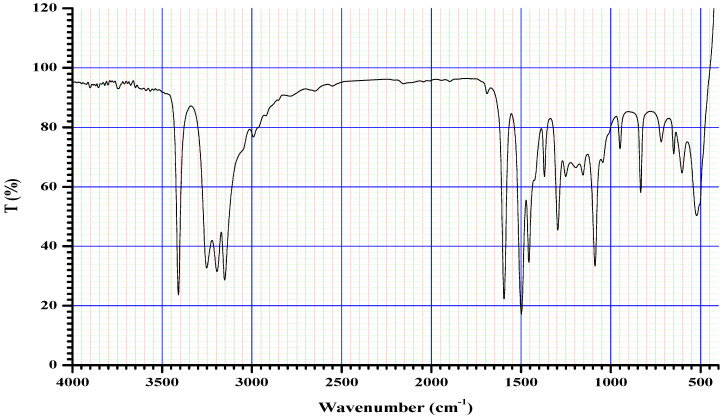
Infrared spectrum of the ligand thiosemicarbazone (H_2_L).

**Figure 12:**
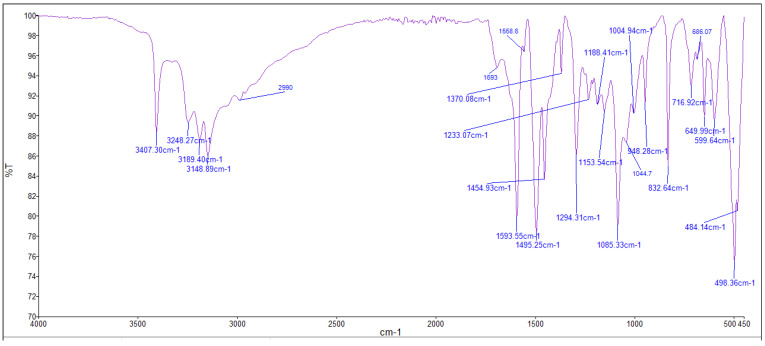
Infrared spectrum of the Co^+2^ complex.

**Figure 13:**
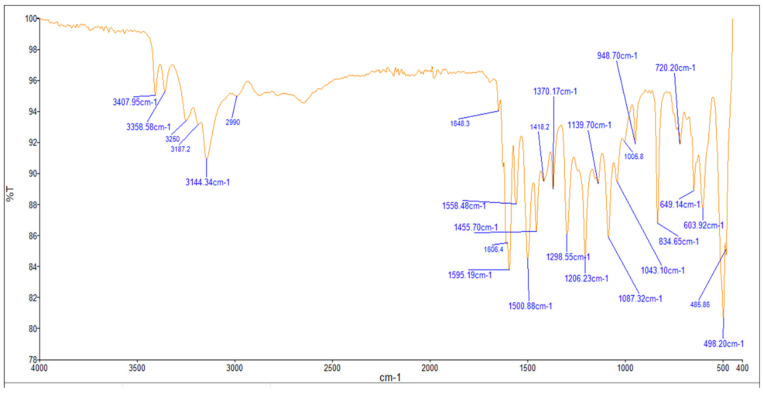
Infrared spectrum of the Cu^+2^ complex.

**Figure 14:**
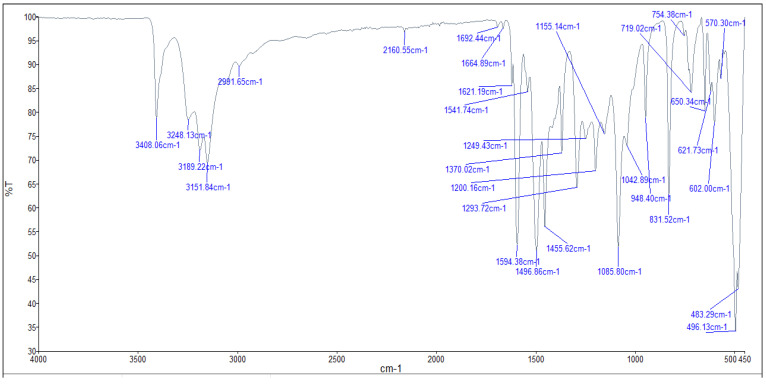
Infrared spectrum of the Zn^+2^ complex.

The FTIR bands of the thiosemicarbazone ligand spectrum (Figure 11) showed peaks at 3408, 3248, 3194, and 3149 cm^−1^, which could be attributed to the imine ν(N-H) and ν(NH_2_) [47,48,49]. The weak bands appearing at 3000, 2950, and 2990 cm^−1^ could be attributed to the azomethine and aliphatic protons [50,51]. The spectrum also displayed bands at 1594 and 833 cm^−1^, which are related to imine ν(C=N) and thioamide ν(C=S) [52,53] groups, respectively (Table 3). These observations indicate that the thiosemicarbazone existed only in thioketo form in the solid state. This conclusion is endorsed by the absence of a band related to the ν(S–H) linkage (generally appearing in the range of 2500–2600 cm^−1^) [43,44,45,54,55]. The manner of coordination can be observed by matching the FTIR spectra of the complexes shown in Figure 12, Figure 13 and Figure 14 with the FTIR spectrum of the thiosemicarbazone (H_2_L). Matching clarifies that the ligand (H_2_L) functions as a dibasic tetradentate, bonding with the Cu^2+^ and Co^2+^ ions via the imine nitrogen atom. The thioamide group, in its thioenol form, in the Cu^2+^ and Co^2+^ complexes forms a tetradentate structure, and this confirmed, from the FTIR spectroscopy, binding of nitrogen atoms by observing shifts in the C=N stretching frequency to lower wavenumbers. This method of chelation was supported by the following four proofs:

i. The negative shift in position (4–30 cm^−1^) and intensity of the band of the imine group C=N) [56].
ii. The displacement of the imine group ν(N-H6,7) band upon chelation, signifying that the thiosemicarbazone in these complexes interacted in the thioenol form, that was endorsed by the appearance of a new band in the 1640–1690 cm^−1^ region, assignable to ν(C=N-N=C) [56].
iii. The negative shift in position (113–184 cm^−1^) and intensity of the ν(C=S) group, which appears in the 649–720 cm^−1^ range due to the ν(C-S) group [57,58].
iv. The appearance of new bands at 451–476 and 497–519 cm^−1^, which could be attributed to ν(M-S) and ν(M ← N), respectively [58].

On the other hand, the ligand (H_2_L) worked as a neutral tetradentate, bonding with the Zn^2+^ ion via the imine nitrogen atom and the thioamide group in its thioketo form. It is important to note that in the spectrum, the region around 3400–3100 cm^−1^ shows a broad, deep absorption, which indicates overlapping N–H stretching vibrations (possibly two N–H groups from the thiosemicarbazone moiety). Even though it is labeled individually, it is part of the broad, strong N–H/O–H stretch that absorbs strongly because these vibrations cause large changes in dipole moments. N–H and O–H bonds are quite polar. Their stretching motions produce a large change in dipole moments, which leads to high IR absorption intensity. That is why even when the peaks are broad and overlapping, they are still classified as strong.

### 2.5. Thermal Gravimetric Analysis (TGA)

**Figure 15:**
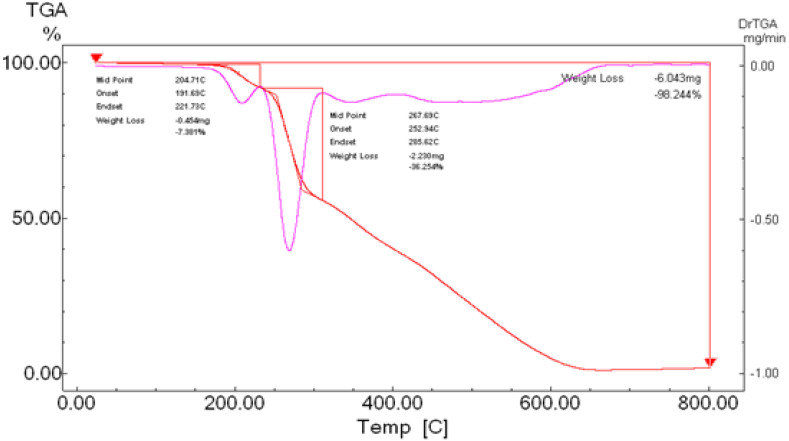
TGA spectrum of the ligand thiosemicarbazone.

**Figure 16:**
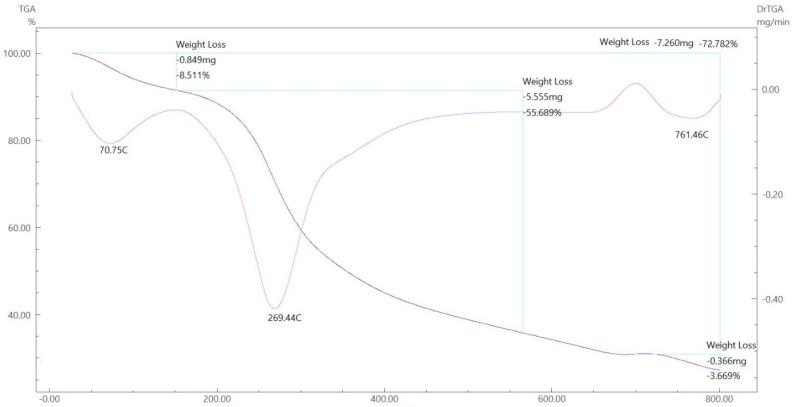
TGA spectrum of the Co–ligand complex.

**Figure 17:**
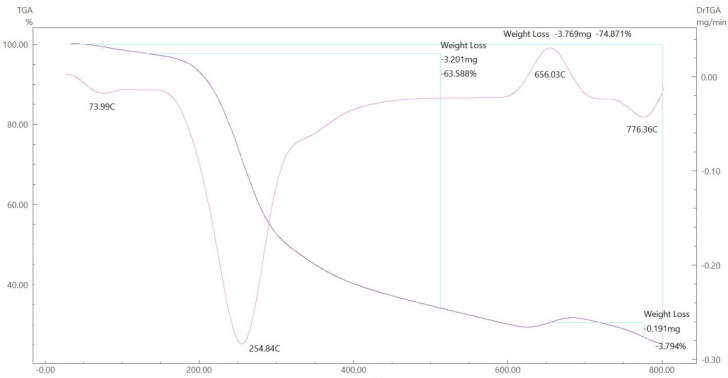
TGA spectrum of the Cu–ligand complex.

**Figure 18:**
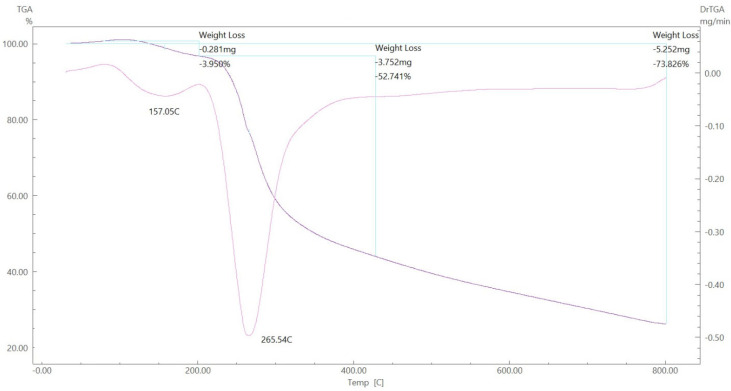
TGA spectrum of the Zn–ligand complex.

To gain further insight into the nature of the complexes and their stabilization properties, thermo-gravimetric analysis was performed in the 25 to 800 °C range. The data are summarized in Table 4, and as shown in Figure 15, Figure 16, Figure 17 and Figure 18, it is evident that there is a clear correlation between the weight loss achieved using the suggested and calculated formulae. The TG thermogram showed that the ligand is stable up to 191 °C and loses the amino group in the temperature range of 191–221 °C, subsequently decomposing completely in the temperature range of 230–650 °C with a weight loss of 91.67% (calculated: 93.10%) (Figure 15). The Co^2+^and Cu^2+^ complexes displayed weight losses between 70 and 73 °C and referred to two water molecules beyond the coordination sphere. The Co^2+^ and Cu^2+^ complexes decomposed in one step within the temperature range of 250 to 800 °C, with weight losses of 63.58% and 64.19% (calculated at 62.53% and 63.41%), leaving Cu^+2^C and Co^+2^C, as shown in Figure 16 and Figure 17. The TG thermogram for the Zn^2+^ complex showed that it is stable up to 157 °C and then starts to decompose to eliminate C1_2_H_26_N_12_S_2_ at 265 to 800 °C, with a weight loss of 73.83% (calculated: 75.56%), leaving Zn_2_S as a residue, as shown in Figure 18 [59,60].

### 2.6. Differential Scanning Calorimetry (DSC) Characterization

**Figure 19:**
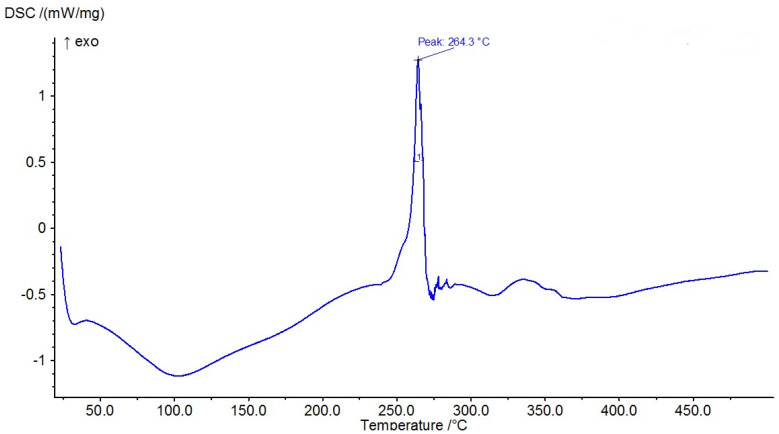
TG thermogram of the Co^+2^ complex.

**Figure 20:**
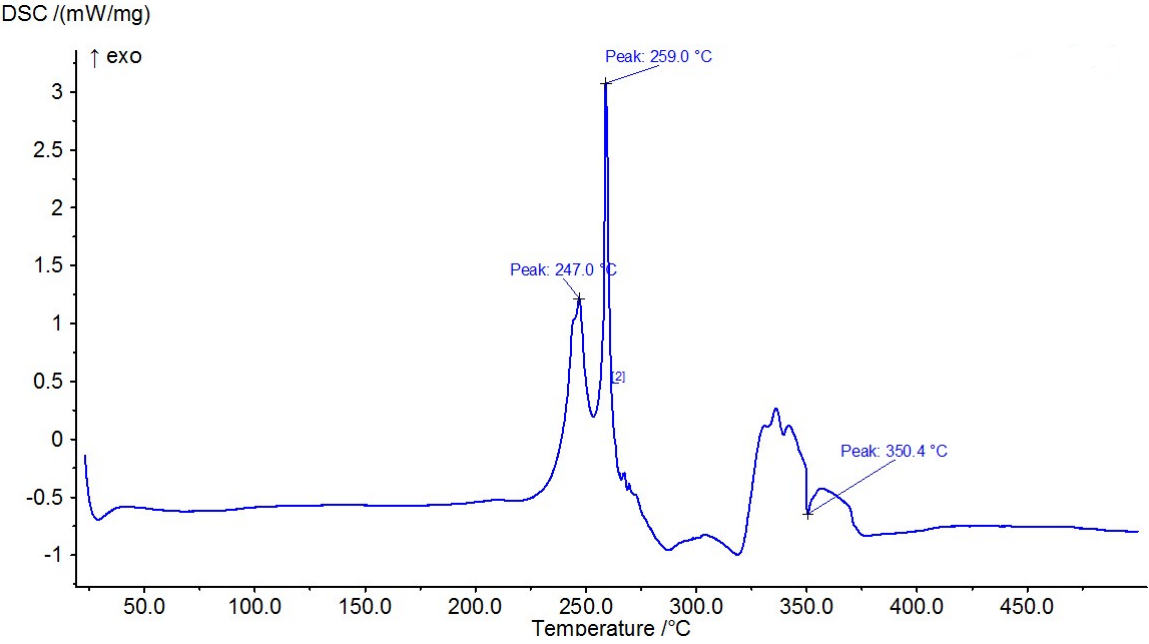
TG thermogram of the Cu^+2^ complex.

**Figure 21:**
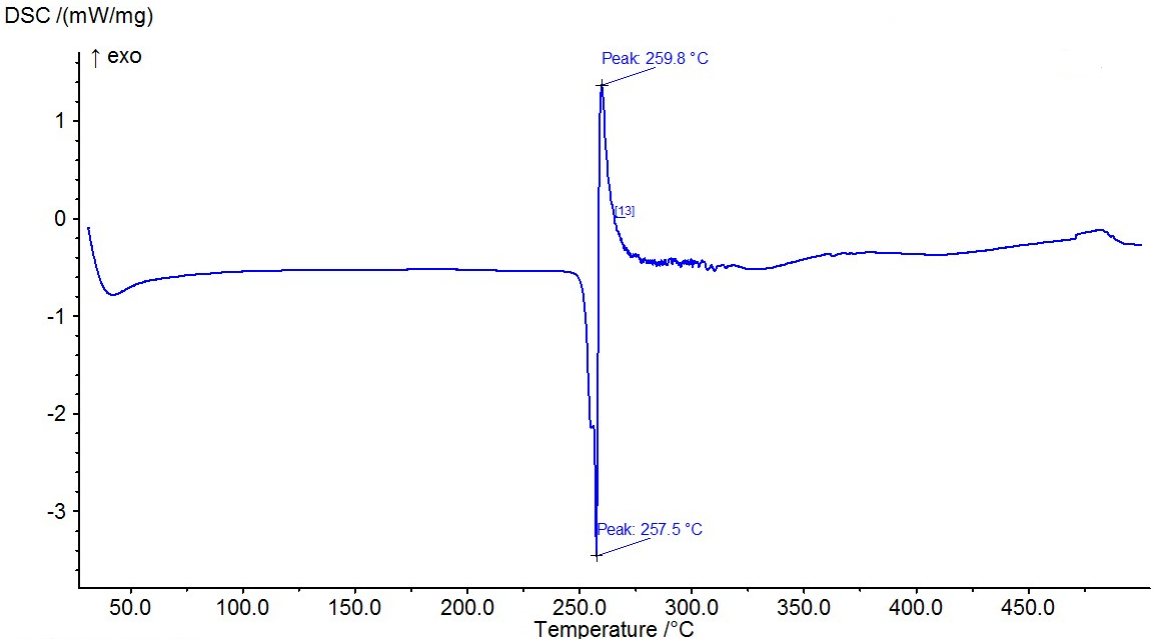
TG thermogram of the Zn^+2^ complex.

The results of the measured DSC curves for the Co, Cu, and Zn complexes show a melting point range between 50 °C and 450 °C, as plotted in Figure 19, Figure 20 and Figure 21. The metal complexes showed different behaviors, with sharp endothermic peaks at the 200–350 °C range first, which corresponds to the metal complexes’ melting points, and then broad exothermic peaks in the Co^+2^ and Cu^+2^ complexes at 350–450 °C, which represent their decomposition to form the respective metal sulfides. In the Co–ligand complex, the decomposition peak was observed at 264 °C (Figure 19). In the Cu–ligand complex, three peaks were observed at 247.0 °C, 259.0 °C, and 350.4 °C (Figure 20). Meanwhile, in the Zn–ligand complex, two peaks were observed at 257.5 °C and 259 °C (Figure 21). In depth, the first complex to melt was the Cu–ligand complex, followed by the Zn–ligand complex, and lastly, the Co–ligand complex as the more difficult complex to melt.

### 2.7. Investigation of the Complexes’ Morphology by SEM

The surface morphology of the metal complexes was assessed, and several micrograph images were taken at different magnifications, as shown in Table 5. The morphological characterization of the Co, Cu, and Zn complexes revealed smooth nanoparticle shapes without pores or cracks but with sharp edges. Generally, the nanoparticles’ size is better than the micro size [52].

### 2.8. Particle Size Measurements of Metal–Ligand Complexes by DSC

The particle sizes of the metal complexes are summarized in Table 6. The data show that all metal complexes were in the nano-sized range, with diameters ranging from 226 to 266 nm. The Cu complex exhibited the smallest particle size, followed by the Zn complex, and then the Co complex.

### 2.9. Cell Proliferation Assessments

The MTT assay of the metal complexes was tested against a breast cancer cell line (MDA-MB-453). The data were obtained at different concentrations (0, 2.5, 5, 10, and 20 µM) of the metal complexes, and the results are summarized in Table 7. According to a biological evaluation, no detectable toxicity was observed prior to the 48 h treatment period [37,59,60], and cancer cell proliferation was inhibited for at least 72 h. The findings reveal that metal–ligand complexes possess strong anticancer properties. In depth, the Cu–L complex was the most potent agent, killing MDA-MB-453 cells, with an IC_50_ value of 3.31 ± 0.34 µM. The second potent agent was the Zn–L complex, followed by the Co–L complex, with IC_50_ values of 32.1 ± 4.80 and 48.1 ± 3.06, respectively. The synthesized complexes proved their strength as anticancer agents in comparison with the healthy HEK293 cells that exhibited high IC_50_ values and did not cause strong cell death. Studies show that for the clinically proven drug cisplatin, its IC_50_ values, measured by the MTT assay to determine cell death, vary significantly depending on the cancer cell line, treatment duration, and the presence of other compounds. For instance, a study on Hela cells reported an IC_50_ of 8.93 µM, while a study on MCF-7 cells showed an IC_50_ of 7.5 µg/mL for cisplatin, and another found an IC_50_ of 7.49 µM for A549 cells. Also, in ovarian and prostate cancer models, the IC_50_ was around 10–50 µM. These results support our data demonstrating that the Cu–complex is a potent anticancer agent [61,62,63] compared to the drug cisplatin.

### 2.10. Computational Analysis of Optimized Geometry and Energy Gap

**Figure 22:**
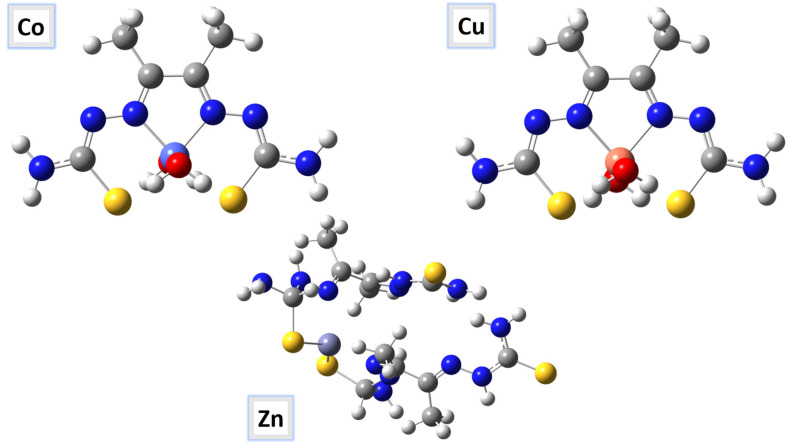
The optimized molecular structures of the Co–, Cu–, and Zn–ligand complexes are shown as ball-and-stick models.

**Figure 23:**
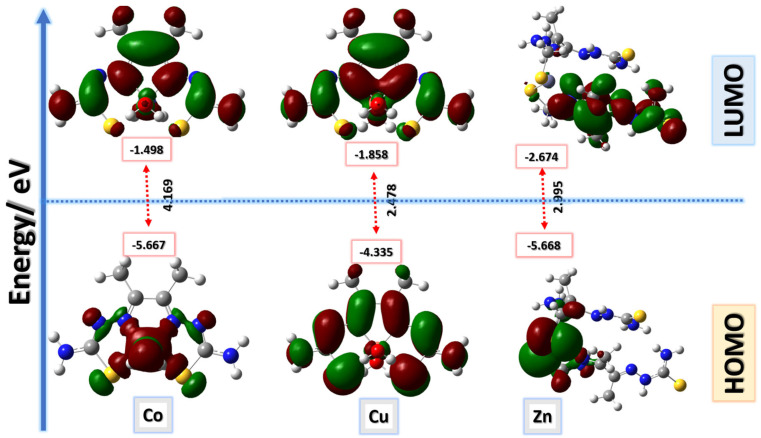
Frontier molecular orbitals for Co–, Cu–, and Zn–ligand complexes calculated in the gas phase using the B3LYP/GENECP method; energy level of HOMOs and LUMOs; and E_gap_ (ΔE).

**Figure 24:**
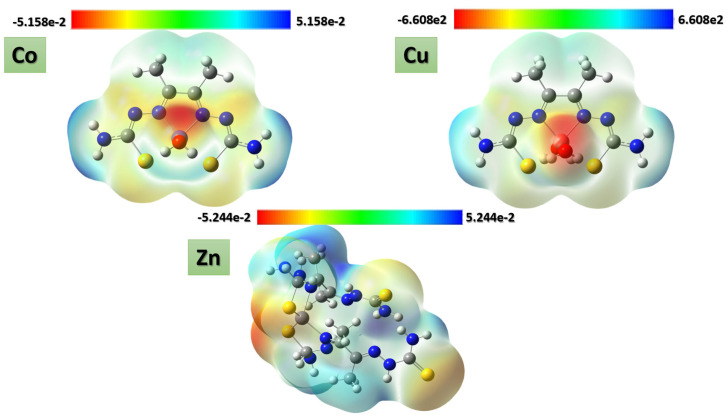
ESP maps of Co–, Cu–, and Zn–ligand complexes by mapping the total density over the molecular electrostatic potential calculated in the gas phase.

Several physical properties, including frontier molecular orbital energies (eV), ionization potential (IE), electron affinity (EA), electronegativity (χ), global electrophilicity (ω), chemical potential (µ), global softness (S), and dipole moment, can be determined by optimizing the structure and frequency of compounds using quantum chemical calculations. For the metal–ligand complexes, the geometric characteristics are summarized in Figure 22, Figure 23 and Figure 24, and the physical parameters are given in Table 8. In this study, the lowest unoccupied molecular orbital (LUMO), which acts as an electron donor; the highest occupied molecular orbital (HOMO), which acts as an electron acceptor; and the structural parameters, dipole moments, and nonlinear optical properties of the Co, Cu, and Zn complexes were calculated by both B3LYP/LANL2DZ and B3LYP/GENECP/LANL2DZ-6-311G (B3LYP/MIX) methods. In the optimized structures of the complexes, the metal salt was found to be complexed with two water molecules, as confirmed by various spectroscopic tools. The frontier molecular orbital HOMO of the complexes exhibits similar behavior, and the charge density localizes in the metallic region of all complexes. The energy gap (ΔEHOMO-LUMO) is the difference in energy between the HOMO and LUMO (Figure 23) [64,65]. A molecule is more stable, harder, and less reactive when its energy gap (ΔE) is larger, and vice versa [65]. Therefore, the order of the energy gap values is arranged in the following order: Co > Zn > Cu complex. The calculation of the Cu–ligand complex proved that it is the least hard complex (η) with the highest softness (σ) parameter, which proved that this complex is the most reactive complex biologically, followed by the Zn and then Co complexes. The DFT calculation agreed with the biological anticancer results. Figure 24 illustrates the electrostatic potential maps in the three-dimensional distribution of charge in each metal–ligand complex, represented by several colors: the most electronegative is represented by red, the most positive is represented by blue, and zero potential is represented by green [66]. Red < orange < yellow < green < blue is the order in which the potential progressively rises from negative to positive. The lone pair of electronegative atoms is typically found in regions with the highest electronegative potential. From the MEP map of the metal–ligand complexes, it can be observed that the negative regions in the molecule are considered as nucleophiles, such as the oxygen, sulfur, and nitrogen atoms of the complex. However, the maximum positive regions are localized around the electrophile’s hydrogen atoms, and these findings are supported by previous studies [67,68].

### 2.11. Antibacterial Evaluation

The antibacterial activity of the newly synthesized free ligand and the Co–, Cu–, Zn–ligand complexes was evaluated using the disk diffusion method against Gram-positive (*Bacillus subtilis*) and Gram-negative bacteria (*E. coli*). Trials were conducted three times, and the recorded values represent the mean average, as shown in Table 9. The results show that the free ligand was ineffective against both Gram-positive and Gram-negative bacteria, as proven in a study on the chelating activity of (2E,2′E)-2,2′-(pyridine-2,6-diylbis(ethan-1-ylidene)) bis(N-ethylhydrazinecarbothioamide [69]. In Gram-positive bacteria, the most potent nano metal–ligand complex was copper, followed by the cobalt complex, with no effect on bacterial growth inhibition observed for the zinc complex. This is consistent with chelation theory, which postulates that chelation enhances metal complexes’ capacity to cross the cell membrane and explains the antibacterial action of metal complexes [70,71,72]. In our study, the Cu-L complex had more potent inhibition zones compared to the bacterial drugs penicillin and ampicillin [73], with 6 and 7mm, respectively, against Gram-positive and negative bacteria [69]. On the other hand, the data show a weak effect against Gram-negative bacteria at different tested concentrations (5 and 10 mg), with no effect observed for the other complexes [69]. A study revealed that nano Co– and Zn–ligand complexes showed antibacterial activity that is higher than the free ligand. In sum, according to the findings, when compared to the free ligand, the copper complex shows promise as an antibacterial agent in Gram-positive and negative bacteria.

## 3. Discussion

In this research, we designed and synthesized metal–ligand complexes and biologically evaluated them. The physical parameters and chemical characterization confirmed the complexes’ molecular weight, purity, and structures. The TGA and DSC results are parallel and prove the complexes’ stability under the effect of different temperatures. According to the TGA data, the complexes were stable until 70 °C, while according to the DSC data, the complexes’ stability reached 247 °C. It is essential to note that the normal body temperature is 37 °C, and a fever ranges from 38 °C to 41 °C, which indicates that our complexes are stable if they are considered as chemotherapy drugs in the future.

The results of the SEM and DLS techniques are parallel and prove the nanoparticles’ morphologies and the sizes of the complexes. According to previous research, nanoparticles readily accumulate in cancer cells [73,74,75]. Furthermore, these complexes are excellent choices for delivering anticancer medications to cancer tumors and tissues due to their small-sized nanoparticles and biocompatibility [76]. Studies have proven that many nanoparticles are selective, promising agents for anticancer, antimicrobial, and anti-Alzheimer treatments, such as ZnO, AgNPs, SnO, CuO, and FeO [77,78,79,80,81]. Generally, due to their better efficacy and capacity for selective targeting, nanoparticles are beginning to replace more conventional cancer treatments, including radiation, chemotherapy, and surgery [81]. Additionally, studies have proven that the size of nanoparticles determines how they interact biologically with cells and the body, affecting uptake, targeting, and the enhanced permeability and retention effect [52,82,83]. Current chemotherapy for cancer causes cytotoxicity, a lack of selectivity, multidrug resistance, and the growth of stem-like cells [53]. For a range of cancer treatments, these nanomaterials, which target the immune system, DNA, tumor microenvironment, and cancer cells, have been modified to increase drug capacity and bioavailability, reduce toxicity, and enhance selectivity [2] compared to conventional cancer therapy drugs [75,76]. Scientists are constantly developing novel and effective nanoparticles for drug delivery in medicine [44,45] for imaging and diagnostics [66]. In addition, studies have proven that nano complexes possess antibacterial and antifungal properties [84,85,86,87]. In sum, considering all these findings, further research is necessary to mitigate toxicity and improve clinical outcomes in medication delivery using nanoparticles.

When nano metal complexes interfere with critical metabolic processes, break down vital proteins like enzymes, or cause oxidative stress, they can be harmful to cells. Also, a metal’s toxicity is determined by its type, its concentration, and the cell’s capacity to detoxify or control it. The production of reactive oxygen species (ROS), DNA damage, and disruption of cellular functions, such as differentiation and proliferation, are some of the mechanisms of action. These complexes can stop tumors from growing and spreading by disrupting their growth. However, toxicity, resistance, and selectivity issues frequently plague the research and therapeutic application of metal compounds, despite their potential as anticancer medicines [88,89,90,91]. Additionally, while considering metal complexes with antibacterial properties, several elements should be considered, including the chelate effect, the ligands’ nature, the complex’s overall charge, and the type of ion that neutralizes the specific ionic complex [92]. The findings of a recent 2023 study support our finding that the copper complex exhibited superior anticancer activity and selectivity against a cisplatin-resistant lung cancer cell line [91]. Previously, studies showed that thiosemicarbazone complexes have antimicrobial [17,19], antiviral [4], antibacterial [25,37,38], antifungal [4], and anticancer activities [25,37,38]. Additionally, research has shown that metal complexes have stronger anticancer effects than free ligands [54], which is parallel to our anticancer data. All these data are supported by a study conducted by Hoda El-Shafiy in 2017, where the nano complexes of Zn (II), Cu (II), Ni (II), and Co (II) ions were much more potent than the ligand itself [92].

The coordination, orientation, aromatic moiety, ligand binding [3], and solubility of metal ions all contribute to the overall stability of such complexes [54]. In addition, practical biomedical research focuses on synthesizing new transition metal complexes at the nanoscale to create new materials with unique physical, chemical, and biological properties [64] that have effective potent biological activities, which can benefit the medical field. Despite being a very successful platinum-based medication, cisplatin has serious adverse effects, including ototoxicity, neurotoxicity, and nephrotoxicity. Alternatives with potentially distinct toxicity profiles or modes of action are being investigated, including complexes with metals including gold, ruthenium, and copper, as well as the platinum analogs carboplatin and oxaliplatin. Furthermore, cisplatin also causes gastrointestinal toxicity, ototoxicity, and myelosuppression. Research is actively focused on developing new metal complexes with better safety profiles and improved efficacy with promising potentials [93,94,95]. Based on all these results, the synthesis and development of new agents is required.

The results of the DFT calculation of the metal–ligand complexes agree with the biological anticancer results. The Cu complex is soft and more biologically active than other complexes. A study conducted in 2021 by Howsaui et al. found that the Cu(II) complex exhibits good reactivity, revealing good toxicity and inhibition activity against the examined cell lines [96]. In relation to electron density, the molecular electrostatic potential (MEP) is a crucial metric for identifying potential sites of electrophilic/nucleophilic attack and hydrogen-bonding interactions in newly synthesized complexes. These results support our finding that the Cu complex is potent.

Moreover, according to the findings of an antibacterial study, when compared to a free ligand, copper complexes also show promise as antibacterial agents against both Gram-positive and Gram-negative bacteria. The complexes can act by damaging cell walls, killing bacteria, or preventing biofilm formation [97]. The recent findings between in vitro and computational studies will provide a solid background for researchers for future developments.

## 4. Materials and Methods

### 4.1. Materials and Reagents

We synthesized the metal–ligand complexes in our laboratory at Kuwait University. All chemicals and biological supplies were graded as analytical reagents. They were pure and ready for direct use, including hydrazine carbothioamide, ethanol, diethyl ether, DMSO, CuCl_2_, CoCl_2_, ZnCl_2_, the DMEM medium, FBS, penicillin/streptomycin, 1% L-glutathione, PBS, WST-1 test Kit (ab65473, Abcam, Cambridge, MA, USA), and nutrient agar. All were purchased from Sigma-Aldrich, Germany, and E. Merk. The *Escherichia coli* (*E. coli*) and *Bacillus subtilis* strains were provided by the biology department. All complexes were dissolved in dimethyl sulfoxide to prepare chemical stock solutions (μg/mL) for antibacterial treatment and assessment. In addition, all bacterial studies were performed near a flame to prevent any contaminations.

### 4.2. Instruments Used

All chemical spectral analyses were conducted at the Research Sector Project Unit (RSPU) at Kuwait University. Thin-layer chromatography (TLC) was used on pre-coated aluminum plates with silica gel (Merk 9385) to detect the reaction endpoints. The molecular weights of the samples were determined using the EI (70 eV) mode of the VG AutoSpec QMS 30 and MSg (AEI) spectrometer and were then confirmed by mass spectroscopy using LCMS. On an elemental uni-cube superuser, elemental analysis (CHNS) was carried out. A Bruker DPX 600 NMR spectrophotometer (Bruker, Billerica, MA, USA) was used to collect ^1^H and ^13^C nuclear magnetic resonance (NMR) spectra at 600 MHz and 150 MHz, respectively, in DMSO and CDCl_3_. Fourier-transform infrared spectroscopy (FTIR) spectra were collected in the 400–4000 cm^−1^ range using a Jasco 6300 FTIR (Jasco, Tokyo, Japan). A scanning electron microscope (SEM) was used to check morphology. The dynamic light scattering (DLS) technique was used to determine the size of the metal complexes (Malvern ZetaSizer (ZS)). Additionally, heat flow was measured using DSC to characterize the metal–ligand complexes. Furthermore, the sample’s weight change in relation to temperature was measured using thermal gravimetric analysis (TGA).

### 4.3. Synthesis

#### 4.3.1. General Synthesis of the Ligand: 2,2′-(butane-2,3-diylidene) Bis(hydrazine-1-carbothioamide)

**Scheme 2:**
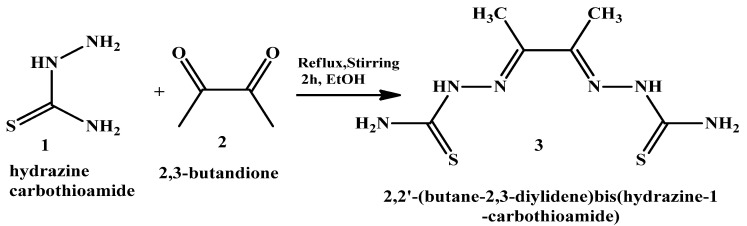
The starting materials hydrazine carbothioamide and 2,3- butandione that were used to synthesize the ligand H_2_L (3).

2,2′-(butane-2,3-diylidene) bis(hydrazine-1-carbothioamide) (H_2_L) (3) was synthesized as shown in Scheme 2 via the gradual addition of 30 mL of a hot ethanol solution of hydrazine carbothioamide (1) (18.12 g, 0.2 mol) to 30 mL of a hot ethanolic solution of 2,3-butandione (2) (23.2 g, 0.1 mol). This was followed by heating the mixture under reflux with continuous stirring, utilizing a heating mantle, for two hours. The formed precipitate was collected by filtration, washed with absolute ethanol and diethyl ether, and finally dried under vacuum over silica gel to produce H_2_L (3) with a yield of 77%.

#### 4.3.2. General Synthesis of the Metal–Ligand Complex

**Scheme 3:**
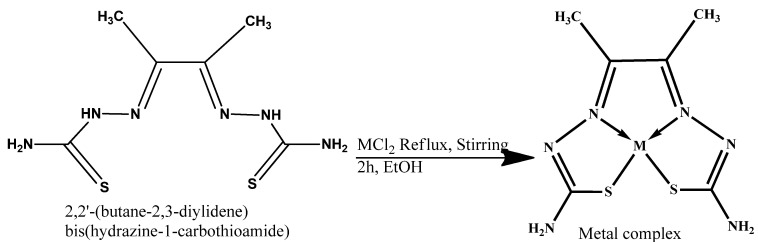
General scheme of chelation of the metal–ligand complexes.

The synthesized 2,2′-(butane-2,3-diylidene) bis(hydrazine-1-carbothioamide) (H_2_L) (3) was used as a ligand (as a Lewis base–electron donor) for the reaction with metal chloride salt (as a Lewis acid–electron acceptor) to form our target complex. The Co–, Cu–, and Zn–complexes were prepared by refluxing 1 mmol of the ligands (3) under investigation with 1 mmol of the metal salt CuCl_2_, ZnCl_2_, and CoCl_2_ in an ethanoic solution in a water bath for 2–3 h, as shown in Scheme 3. The resulting solid metal–ligand complexes were filtered off; washed several times with absolute ethanol, followed by diethyl ether; and then dried in a vacuum desiccator over anhydrous calcium chloride, yielding a product of 80% purity. In addition, to make sure that the complexes were free of Cl ions, we ran a silver nitrate test. First, we dissolved a small amount of the metal–ligand complex in distilled water. Then, we added a few drops of diluted nitric acid (HNO_3_) to remove any interfering anions, like carbonate ion. There were no observable white precipitates of AgCl, which means that there were no free chlorides present in the metal–ligand complexes.

### 4.4. Generating Thermal Gravimetric Curves (TGA)

This technique provided more insights into the stabilization and water molecule characteristics of the complexes. TGA was conducted for all metal–ligand complexes, weighing 5 mg, which were placed in a Pt pan and heated in a nitrogen environment between 25 and 800 °C for 10 min. According to TGA statistics, a clear correlation exists between the calculated and recommended formulas for weight loss [98].

### 4.5. Differential Scanning Calorimetry Technique (DSC)

The thermal analysis tool known as DSC monitors a sample’s temperature and physical properties over time in both hot and cold conditions. Five milligrams of each sample were weighed on a sealed Al pan, heated from 25 to 500 °C for ten minutes at a heating rate in an argon environment, and the DSC curves for the metal–ligand complexes were then recorded [59,60,69].

### 4.6. Investigating the Complexes’ Morphology by SEM

The surface morphology of the complexes was assessed by utilizing the JEOL-EDS-KU-RSPU system to obtain several SEM readings at 15.0 kV at various micrograph magnifications (×100, ×500, ×1000, ×2000, and ×5000). Uniform morphologies were identified through morphological characterizations [74,76].

### 4.7. Particle Size Measurements by DLS

The Malvern Zeta Sizer (ZS) is an analytical instrument used to measure the size, zeta potential, and molecular weight of particles in solutions. It utilizes dynamic light scattering (DLS) for particles with sizes ranging from 0.3 nm to 10 µm. The electrophoretic light scattering (ELS) is >±500 mV. The temperature controls range from 0 °C to 90 °C, are sensitive, and can work with small sample volumes. This technique is ideal for nanoparticle analysis and stability studies in pharmaceuticals and material sciences. All metal complexes were solubilized in DMSO, and readings were measured ± standard deviations [74,75].

### 4.8. Cytotoxicity Assessment and Statistical Analysis

MDA-MB-453 is a human breast cancer cell line, which is employed in in vitro screening investigations. The cells were cultured in 5% CO_2_ humidified air with 10% FBS, 1% penicillin/streptomycin, and 1% L-glutathione added in the DMEM medium. Before use, fetal bovine serum (FBS) was inactivated for 30 min at 37 °C in a water bath. The manufacturer’s protocol of the WST-1 assays, Kit (ab65473 Abcam, USA) was followed to conduct an in vitro cell proliferation assay. To put it briefly, after seeding, on day one, the cells were exposed to the metal–ligand complexes for 72 h while they were between 75% and 85% confluent. Subsequently, each well received 10 µL of the WST-1 reagent. Using a plate reader (SoftMax Pro 9.0 Flex Station, Molecular Devices, San Jose, CA, USA), the absorbance at 440 nm was measured to calculate the amount of formazan generated. There were four duplicates of each concentration used in the studies. Statistical analysis was performed using GraphPad Prism 09. Every in vitro datapoint is displayed as the average ± standard error. For statistical significance, *p* values ˂ 0.05 were used [58,59,99,100].

### 4.9. Molecular Geometry and Computational Methodology

All computations were conducted using the Gaussian 16 program [60]. The molecular geometry for the studied compounds was fully optimized using the density functional theory B3LYP method. B3 stands for Becke’s three-parameter exact exchange functional combined with the gradient-corrected correlation functional of Lee, Yang, and Parr (LYP) [47,48,49,50], and the DFT/GENECP level was obtained by implementing Def2TZVP. No symmetry constraints were applied during geometry optimization. The choice of a mixed basis set was due to the flexibility, accuracy, consistency, and better performance of Alrich’s effective core potential basis set (def2-TZVP) with a Gaussian-type triple-ζ potential (6-311++G(d,p)). By using HOMO and LUMO energy values for the metal–ligand complexes, electronegativity and chemical hardness could be calculated as follows: X = (I + A)/2 (electronegativity), η = (I − A)/2 (chemical hardness), and S = 1/2η (chemical softness), where I and A are ionization potential and electron affinity, I = EHOMO, and A = ELUMO. NBO calculations were performed at the same level of calculation using the NBO 3.1 program, as implemented in the Gaussian 09W software package. Throughout this study, optimized structures were visualized using the Chemcraft version 1.6 package and GaussView version 5.0.9 [101,102,103,104,105,106,107,108].

### 4.10. In Vitro Antibacterial Activity

As directed by Difco, pure culture plates were prepared two days prior to use. A bacterial suspension was prepared using a nutrient broth, and the bacterial culture was incubated at 37 ± 1 °C. A few milliliters of the bacterial suspension from each strain was added to 20 mL of fresh broth, resulting in a final concentration of approximately 5 × 10^5^ CFU/mL. A suspension containing 0.2 mL of each of the investigated compounds (5 and 10 mg/mL) was cultured for 24 h. In nutrient agar media (NA), the strains of the microorganisms were uniformly distributed. The studied samples were placed inside disks of Whatman paper, which had a diameter of 5 mm. After the socked disks were placed on the NA, the diameters (in millimeters) of the clear inhibition zones encircling the sample were measured using a ruler to determine the inhibition power against those specific organisms. Using the agar disk diffusion method with the minimum inhibitory concentration, the metal–ligand complexes were administered in triplicate to both Gram-positive and Gram-negative bacteria for 24 h to test for antibacterial activity. To avoid contamination, all experimental work was conducted close to a flame. Four mm wells were created using a cork borer, and 100 μL of the metal–ligand complex was added to each well by a micropipette and incubated at 37 °C overnight. Three replicas were used to assess the inhibition zones surrounding the wells, and results are reported as the average. To compare the potency and importance of metal–ligand complexes as antibacterial agents, a free ligand was used as a reference control in the current study [51,109,110].

## 5. Conclusions

Over the past few decades, Co(II), Cu(II), and Zn(II) complexes have gained increasing popularity in chemical biology, particularly as powerful sensory tools and novel antibacterial and anticancer agents. Following their synthesis, a range of analytical methods were employed to characterize and thoroughly describe three novel metal–ligand complexes. These nanoscale complexes demonstrated stable and strong anticancer activity in a breast cancer cell line. Theoretical DFT calculations yielded results equivalent to those observed in cell proliferation. The metal–ligand complexes exhibited a strong antibacterial action against both Gram-positive and Gram-negative bacteria, as indicated by an antibacterial study. In general, the Cu–ligand complex exhibited the strongest antibacterial and anticancer properties, followed by the Zn and then Co complexes. Therefore, the Cu-L complex is promising for developing future therapeutics.

## Figures and Tables

**Table 1:** The analytical and physical characteristics of the ligand H_2_L and its metal complexes.

Compounds	Color	M.Wt.	Dipole Moment μ	M.P. (°C)	Λ^a^	Calculated/(Found) (%)				
						C	H	N	S	M
Free ligand H_2_L (C_6_H_12_N_6_S_2_)	Pale yellow	232.32	--	225	--	31.02 (31.04)	5.21 (5.16)	36.17 (35.07)	27.60 (27.83)	--
Cu complex [Cu(L)].2H_2_O (C_6_H_14_CuN_6_O_2_S_2_)	Dark brown	329.89 (293.97) *	1.249	263–270	4.34	21.84 (21.98)	4.28 (3.80)	25.48 (24.33)	19.44 (18.95)	20.31 (19.89)
Cobalt complex [Co(L)].2H_2_O (C_6_H_14_CoN_6_O_2_S_2_)	Brown	325.28 (288.97) *	1.429	245–248	5.83	22.15 (22.22)	4.34 (4.65)	25.84 (24.70)	19.72 (19.68)	31.60 (31.28)
Zinc complex [Zn(H_2_L)_2_] (C_12_H_26_N_12_S_4_Zn)	Beige	532.06 (535.6)	1.837	268–272	21.52	27.09 (27.83)	4.93 (4.95)	31.59 (31.77)	24.11 (25.38)	19.26 (18.95)

Λ^a^ is molar conductivity in Ω^−1^ cm^2^ mol^−1^. * Complex bound to water (humidity). CHNS is average of three runs.

**Table 2:** ^1^H and ^13^C assignments of the ligand [H_2_L] and Zn^+2^ complex.

Position	H_2_L		[Zn(H_2_L)_2_] Diamagnetic	
	^1^H δ (ppm)	^13^C δ (ppm)	^1^H δ (ppm)	^13^C δ (ppm)
1	--	148.37	--	148.27
2	--		--	
3	1.947	13.81	2.168	11.54
4	--	--	--	--
5	--	--	--	--
6	10.197, 11.061	--	10.208	--
7		--		--
8	--	183.12	--	178.84
9	--		--	
10	8.390, 8.101	--	7.850, 8.404	--
12		--		--
13	--	--	--	--
14	1.947	11.55	2.508	11.54
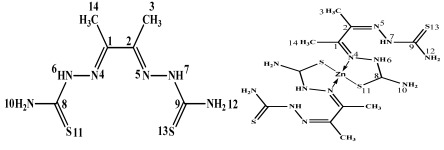				

**Table 3:** FTIR spectral data for H_2_L and its metal complexes and their assignments.

Compounds	ν(H_2_O) ν(NH/NH_2_) cm^−1^	ν(C=N) ν(C=N) * cm^−1^	ν(C=S) ν(C-S) cm^−1^	ν(N-N) cm^−1^	ν(M-N) cm^−1^	ν(M-S) cm^−1^
H_2_L (C_6_H_12_N_6_S_2_)	3408 sbr, 3248 sbr, 3194 sbr, 3149 sbr	1594 s --	833 m --	1300 m	--	--
[Cu(L)].2H_2_O (C_6_H_14_CuN_6_O_2_S_2_)	3407 sbr, 3250 sbr, 3187 sbr, 3144 sbr	1595 s, 1648 w	-- 649 w	1298 m	603 m	485 w
[Co(L)].2H_2_O (C_6_H_14_CoN_6_O_2_S_2_)	3407 sbr, 3248 sbr, 3189 sbr, 3148 sbr	1593 s, 1693 w	-- 948m	1294 m	599 m	484 w
[Zn(H_2_L)_2_] (C_12_H_26_N_12_S_4_Zn)	3408 sbr, 3248 sbr, 3189 sbr, 3151 sbr	1594 s --	831 m --	1293 m	602 w	483 w

* New peaks in the structure. The intensity of the peaks: s = strong, w = weak, m = medium, br = broad, sh = sharp.

**Table 4:** Thermo-gravimetric data of thiosemicarbazone and its complexes (the minimum temperature = 25 °C and maximum temperature = 800 °C).

Compounds	Temp. (°C)	Weight Loss (%) Found (Calcd.)	Assignment
Free ligand H_2_L (C_6_H_12_N_6_S_2_)	30–191 191–221 230–650	-- 7.38(6.90) 91.67 (93.10)	Stable Elimination of NH_2_ Complete decomposition of the ligand
Copper complex [Cu(L)].2H_2_O (C_6_H_14_CuN_6_O_2_S_2_)	73.99 254–776 800	11.28 (10.91) 63.58 (62.53) 25.13 (26.54)	- 2H_2_O - C_4_H_10_N_6_S_2_ Cu^+ 2^C (Residue)
Cobalt complex [Co(L)].2H_2_O (C_6_H_14_CoN_6_O_2_S_2_)	70 269–761 800	08.51 (11.07) 64.19 (63.41) 27.22 (25.50)	- 2H_2_O - C_4_H_10_N_6_S_2_ Co^+ 2^C (Residue)
Zinc complex [Zn(H_2_L)_2_] (C_12_H_26_N_12_S_4_Zn)	30–157 265–750 800	-- 73.83 (75.56) 26.17 (24.34)	Stable - C_12_H_26_N_12_S_2_ Zn^+2^S (Residue)

**Table 5:** The SEM morphologies of metal complexes at different micrograph magnifications (×100, ×500, and ×1000) at low concentrations (5 and 10 µM) and high concentrations (50 and 100 µM).

Mag.	Co–Ligand Complex	Cu–Ligand Complex	Zn–Ligand Complex
×100	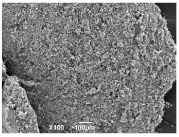	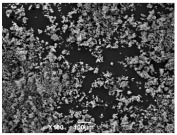	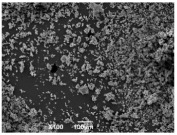
×500	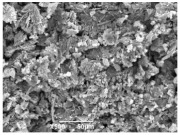	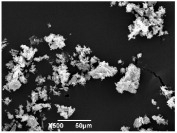	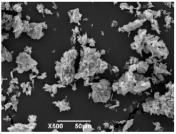
×1000	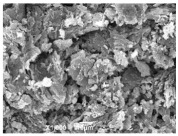	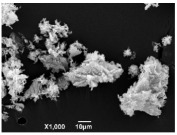	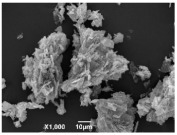
×2000	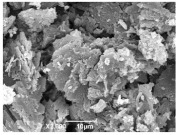	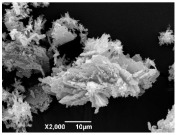	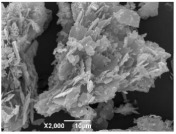
×5000	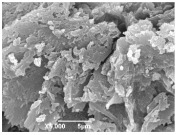	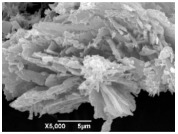	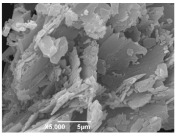

×: different SEM magnifications. Low magnification (×100), medium magnification (×500), and high magnification (×1000).

**Table 6:** Particle size measurements of the synthesized metal–ligand complexes.

**Metal Complex**	**Particle Size ± Standard Deviation** **(d.nm)**	**Graph**	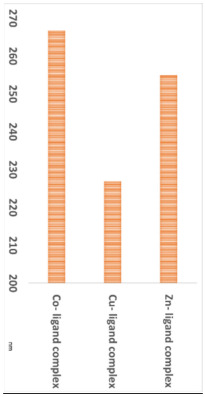
Co–ligand complex	266.7 ± 83.31	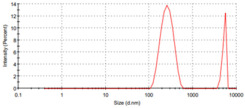	
Cu–ligand complex	226.9 ± 75.18	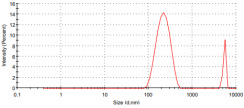	
Zn–ligand complex	254.9 ± 68.87	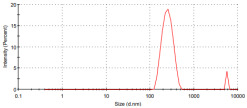	

**Table 7:** IC_50_ data of breast cancer cell line treated with metal–ligand complexes.

Parameter	Dilution Used to Treat the Cells with the Metal Complexes/µg				
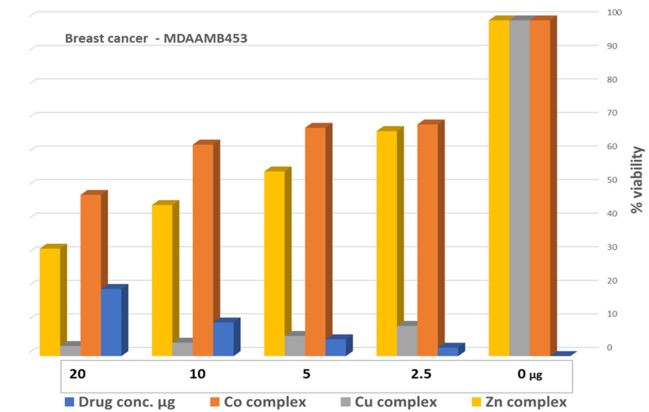					
Co–ligand complex 1:1	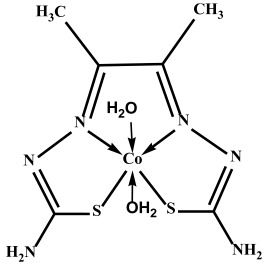 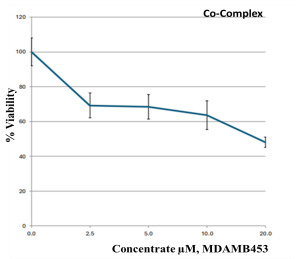				
Drug conc µg	**0**	**2.5**	**5**	**10**	**20**
Mean—blank	100	69.2	68.4	63.6	48.1
Standard deviation	8.00	7.15	7.08	8.29	3.07
HEK293	-	-	-	-	80.50 ± 3.139
Cu–ligand complex 1:1	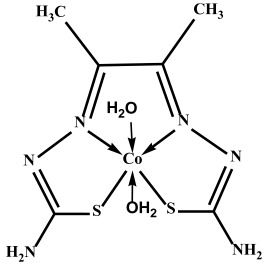 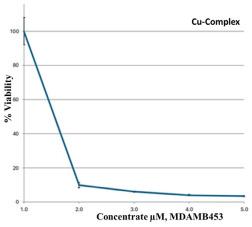				
Drug conc µg	**0**	**2.5**	**5**	**10**	**20**
Mean—blank	100	9.80	6.06	3.91	3.31
Standard deviation	7.97	1.58	0.34	0.44	0.34
HEK293	-	-	-	-	66.66 ± 4.586
Zn–ligand complex 2:1	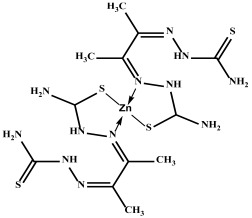 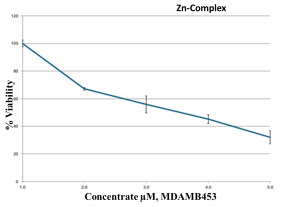				
Drug conc µg	**0**	**2.5**	**5**	**10**	**20**
Mean—blank	100	67.1	55.7	45.2	32.1
Standard deviation	2.45	0.92	6.14	3.17	4.80
HEK293	-	-	-	-	67.77 ± 5.875

MDA-MB-453: breast cancer cell line with high androgen expression. HEK293 is a human embryonic kidney cell used as a healthy control ± standard deviations.

**Table 8:** Theoretical energy calculations and dipole moment of the studied metal–ligand complexes and their interaction products.

ID	E_total_ (Hartree)	E * _HOMO_	E_LUMO_	ΔE	I = −E HOMO	A = −E LUMO	H = (E_HOMO_ − E_LUMO_)\2	S = 1\η	µ = −(1 + A)\2	X = (I + A)/2
Co-L complex	−1175.560	−5.667	−1.498	4.169	5.667	1.498	−2.084	−0.480	−1.249	3.582
Cu-L complex	−1918.132	−4.335	−1.858	2.478	4.335	1.858	−1.239	−0.807	−1.429	3.097
Zn-L complex	−1762.038	−5.668	−2.674	2.995	5.668	2.674	−1.497	−0.668	−1.837	4.171

* E = energy (eV); the lowest unoccupied molecular orbital energy (E_LUMO_); the difference between HOMO and LUMO energy levels (ΔE); Mulliken electronegativity (X); global hardness (η); global softness (S); dipole moment (µ).

**Table 9:** Antibacterial activity of synthesized nano metal–ligand complexes (5 and 10 mg/mL) with the test organisms *E. coli* and *Bacillus subtills*. The drugs penicillin and ampicillin were used as our control for Gram-positive and negative bacteria, respectively.

**Strain/Concentration**	**5 mg**	**10 mg**	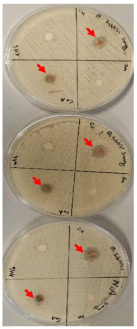
***Bacillus subtills*** **(Gram positive)**	Inhibition zone mm		
Co-L complex	9	9.3	
Cu-L complex	12.3	14	
Zn-L Complex	Nill	Nill	
Free ligand	Nill	Nill	
**Strain/concentration**	**5 mg**	**10 mg**	
***E. coli*** **(Gram negative)**	inhibition zone mm		
Co-L complex	Nill	Nill	
Cu-L complex	5.6	6	
Zn-L Complex	Nill	Nill	
Free ligand	Nill	Nill	
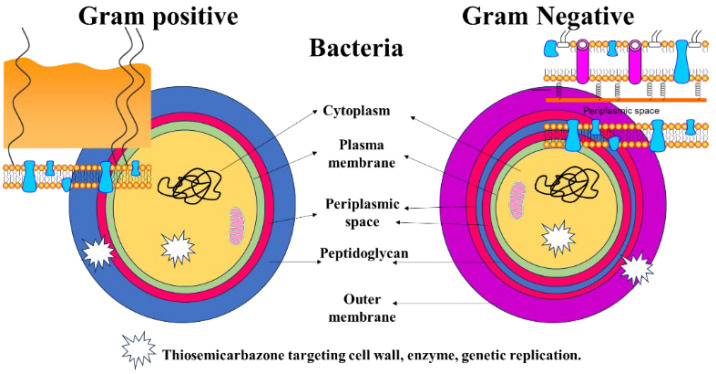			

The zone inhibition diameter in millimeters (mm) and the standard deviation of three separate tests for each strain. Red arrows showed the inhibition zones.

## Data Availability

Data is contained within the article.

## References

[1] Shazia R., Muhammad I., Anwar N., Haji A., Amin A. (2010). Transition metal complexes as potential therapeutic agents. Biotechnol. Mol. Biol. Rev..

[2] Karges J., Stokesm R.W., Cohen S.M. (2021). Metal Complexes for Therapeutic Applications. Trends Chem..

[3] Klaudia J., Marianna M., Suliman Y., Saleh H.A., Eugenie N., Kamil K., Christopher J.R., Marian V. (2022). Essential metals in health and disease. Chem.-Biol. Interact..

[4] Church D.L. (2004). Major factors affecting the emergence and re-emergence of infectious diseases. Clin. Lab. Med..

[5] Van C.R., van de Vijver S., Moore D.A.J. (2017). The global diabetes epidemic: What does it mean for infectious diseases in tropical countries?. Lancet Diabetes Endocrinol..

[6] Ellis T., Eze E., Raimi-Abraham B.T. (2021). Malaria and Cancer: A critical review on the established associations and new perspectives. Infect. Agents Cancer.

[7] Liu Q., Jing W., Kang L., Liu J., Liu M. (2021). Trends of the global, regional and national incidence of malaria in 204 countries from 1990 to 2019 and implications for malaria prevention. J. Travel Med..

[8] Byrne C., Divekar S.D., Storchan G.B., Parodi D.A., Martin M.B. (2013). Metals and Breast Cancer. J. Mammary Gland Biol. Neoplasia.

[9] Maret W. (2016). The Metals in the Biological Periodic System of the Elements: Concepts and Conjectures. Int. J. Mol. Sci..

[10] Maria A., Zoroddu J.A., Guido C., Serenella M., Massimiliano P., Valeria M.N. (2019). The essential metals for humans: A brief overview. J. Inorg. Biochem..

[11] Ejaz H.W., Wang W., Lang M. (2020). Copper Toxicity Links to Pathogenesis of Alzheimer’s Disease and Therapeutics Approaches. Int. J. Mol. Sci..

[12] Pradhan S.H., Liu J.Y., Sayes C.M. (2023). Evaluating Manganese, Zinc, and Copper Metal Toxicity on SH-SY5Y Cells in Establishing an Idiopathic Parkinson’s Disease Model. Int. J. Mol. Sci..

[13] Houtman J.P. (1996). Trace elements and cardiovascular diseases. J. Cardiovasc. Risk.

[14] Górska A., Markiewicz-Gospodarek A., Trubalski M., Żerebiec M., Poleszak J., Markiewicz R. (2024). Assessment of the Impact of Trace Essential Metals on Cancer Development. Int. J. Mol. Sci..

[15] Bjørklund G., Dadar M., Pivina L., Doşa M.D., Semenova Y., Aaseth J. (2020). The Role of Zinc and Copper in Insulin Resistance and Diabetes Mellitus. Curr. Med. Chem..

[16] Raczuk E., Dmochowska B., Samaszko-Fiertek J., Madaj J. (2022). Different Schiff Bases-Structure, Importance and Classification. Molecules.

[17] Mohamed A.A., El-Gedamy M.S., Sadeek S.A., Elshafie H.S. (2025). First Report on some N_2_O_2_-Donor Sets Tetradentate Schiff Base and Its Metal Complexes: Characterization and Antimicrobial Investigation. Chem. Biodivers..

[18] Elshafie H.S., Sadeek S.A., Camele I., Mohamed A.A. (2022). Biochemical Characterization of New Gemifloxacin Schiff Base (GMFX-o-phdn) Metal Complexes and Evaluation of Their Antimicrobial Activity against Some Phyto- or Human Pathogens. Int. J. Mol. Sci..

[19] Mohamed A.A., Ahmed F.M., Zordok W.A., El-Shwiniy W.H., Sadeek S.A., Elshafie H.S. (2022). Novel Enrofloxacin Schiff Base Metal Complexes: Synthesis, Spectroscopic Characterization, Computational Simulation and Antimicrobial Investigation against Some Food and Phyto-Pathogens. Inorganics.

[20] Sousa C., Freire C., De Castro B. (2003). Synthesis and Characterization of Benzo-15-Crown-5 Ethers with Appended N_2_O Schiff Bases. Molecules.

[21] Yildirm L.T., Atakol O. (2002). Crystal structure analysis of Bis{(N,N′-dimethylformamide)-[μ-bis-N,N′-(2-oxybenzyl)-1,3-propanediaminato](μ-asetato) nickel(II)}nickel(II). Cryst. Res. Technol..

[22] Tesauro D. (2022). Metal Complexes in Diagnosis and Therapy. Int. J. Mol. Sci..

[23] Hapke M., Hilt G. (2020). Introduction to cobalt chemistry and catalysis. Cobalt Catalysis in Organic Synthesis: Methods and Reactions.

[24] Valko M., Klement R., Pelikan P., Boca R., Dlhan L., Bottcher A., Elias H., Muller L. (1995). Copper (II) and Cobalt (II) complexes with derivatives of Salen and Tetrahydrosalen: An electron spin resonance, magnetic susceptibility, and quantum chemical study. J. Phys. Chem..

[25] Chang E.L., Simmers C., Knight D.A. (2010). Cobalt Complexes as Antiviral and Antibacterial Agents. Pharmaceuticals.

[26] Sopbué F.E., Songmi F.S., Tamokou J.d.D., Tsopmo A., Doungmo G., Wilhelm P.S.F., Tsamo D.L.F., Ndjakou P.L., Kuiate J.R. (2024). Synthesis, characterization, and antibacterial activity studies of two Co(II) complexes with 2-[(E)-(3-acetyl-4-hydroxyphenyl)diazenyl]-4-(2-hydroxyphenyl)thiophene-3-carboxylic acid as a ligand. BMC Chem..

[27] Kamalakannan P., Venkappayya D. (2002). Synthesis and characterization of cobalt and nickel chelates of 5-dimethylaminomethyl-2-thiouracil and their evaluation as antimicrobial and anticancer agents. J. Inorg. Biochem..

[28] Kaim W., Schwederski B. (1994). Bioinorganic Chemistry: Inorganic Elements in the Chemistry of Life.

[29] Lippard S.J., Berg J.M. (1994). Principles of Bioinorganic Chemistry.

[30] Virag L., Erdodi F., Gergely P. (2016). Bioinorganic Chemistry for Medical Students.

[31] Simunkova M., Lauro P., Jomova K., Hudecova L., Danko M., Alwasel S., Alhazza I.M., Rajcaniova S., Kozovska Z., Kucerova L. (2019). Redox-cycling and intercalating properties of novel mixed copper (II) complexes with non-steroidal anti-inflammatory drugs tolfenamic, mefenamic and flufenamic acids and phenanthroline functionality: Structure, SOD-mimetic activity, interaction with albumin, DNA damage study and anticancer activity. J. Inorg. Biochem..

[32] Olar R., Badea M., Bacalum M., Răileanu M., Ruţă L.L., Farcaşanu I.C., Rostas A.M., Vlaicu I.D., Popa M., Chifiriuc M.C. (2021). Antiproliferative and antibacterial properties of biocompatible copper (II) complexes bearing chelating N,N-heterocycle ligands and potential mechanisms of action. Biometals.

[33] Villarreal W., Castro W., González S., Madamet M., Amalvict R., Pradines B., Navarro M. (2022). Copper (I)-Chloroquine Complexes: Interactions with DNA and Ferriprotoporphyrin, Inhibition of β-Hematin Formation and Relation to Antimalarial Activity. Pharmaceuticals.

[34] Benters J., Flogel U., Schafer T., Leibfritz D., Hechtenberg S., Beyersmann D. (1997). Study of the interactions of cadmium and zinc ions with cellular alcium homoeostasis using 19F-NMR spectroscopy. Biochem. J..

[35] Ye R., Tan C., Chen B., Li R., Mao Z. (2020). Zinc-Containing Metalloenzymes: Inhibition by Metal-Based Anticancer Agents. Front. Chem..

[36] Klug A., Rhodes D. (1987). Zinc fingers: A novel protein fold for nucleic acid recognition. Cold Spring Harbor Symp. Quant. Biol..

[37] Mousa A.B., Moawad R., Abdallah Y., Abdel-Rasheed M., Abdel Zaher A.M. (2023). Zinc Oxide Nanoparticles Promise Anticancer and Antibacterial Activity in Ovarian Cancer. Pharm. Res..

[38] Khashan K.S., Sulaiman G.M., Hussain S.A., Marzoog T.R., Jabir M.S. (2020). Synthesis, Characterization and Evaluation of Anti-bacterial, Anti-parasitic and Anti-cancer Activities of Aluminum-Doped Zinc Oxide Nanoparticles. J. Inorg. Organomet. Polym. Mater..

[39] Bai X.G., Zheng Y., Qi J. (2022). Advances in thiosemicarbazone metal complexes as anti-lung cancer agents. Front. Pharmacol..

[40] Serda M., Kalinowski D.S., Rasko N., Potůčková E., Mrozek-Wilczkiewicz A., Musiol R., Małecki J.G., Sajewicz M., Ratuszna A., Muchowicz A. (2014). Exploring the anti-cancer activity of novel thiosemicarbazones generated through the combination of retro-fragments: Dissection of critical structure-activity relationships. PLoS ONE.

[41] Donmez M., Sekerci M., Adiguzel R., Oğuz E., Türkan F., Yildiko U., Colak N. (2025). Synthesis and characterization of novel bis(thiosemicarbazone) complexes and investigation of their acetylcholinesterase and glutathione S-transferase activities with in silico and in vitro studies. Mol. Divers..

[42] Jain P., Vishvakarma V.K., Singh P., Yadav S., Kumar R., Chandra S., Kumar D., Misra N. (2023). Bioactive Thiosemicarbazone Coordination Metal Complexes: Synthesis, Characterization, Theoretical analysis, Biological Activity, Molecular Docking and ADME analysis. Chem. Biodivers..

[43] Şen B. (2021). 2-Acetyl-5-chloro-thiophene thiosemicarbazone and its nickel(II) and zinc(II) complexes: Hirshfeld surface analysis and Density Functional Theory calculations for molecular geometry, vibrational spectra and HOMO-LUMO studies. Turk. Comput. Theor. Chem..

[44] Gaber M., El-Ghamry H.A., Mansour M.A. (2018). Pd (II) and Pt (II) chalcone complexes. Synthesis, spectral characterization, molecular modeling, biomolecular docking, antimicrobial and antitumor activities. J. Photochem. Photobiol. A Chem..

[45] Ilies D.C., Pahontu E., Shova S., Georgescu R., Stanica N., Olar R., Gulea A., Rosu T. (2014). Synthesis, characterization, crystal structure and antimicrobial activity of copper (II) complexes with a thiosemicarbazone derived from 3-formyl-6-methylchromone. Polyhedron.

[46] Emam S.M., El Sayed I.E.T., Ayad M.I., Hathout H.M.R. (2017). Synthesis, characterization and anticancer activity of new Schiff bases bearing neocryptolepine. J. Mol. Struct..

[47] Chandra S., Bargujar S., Nirwal R., Yadav N. (2013). Synthesis, spectral characterization and biological evaluation of copper(II) and nickel(II) complexes with thiosemicarbazones derived from a bidentate Schiff base. Spectrochim. Acta Part A Mol. Biomol. Spectrosc..

[48] Saghatforoush L.A., Hosseinpour S., Bezpalko M.W., Kassel W.S. (2019). Synthesis, spectroscopic studies and X-ray structure determination of two mononuclear copper complexes derived from the Schiff base ligand N,N-dimethyl-N′-((5-methyl-1H-imidazol-4-yl)methylene)ethane-1,2-diamine. Inorg. Chim. Acta.

[49] Shahsavani E., Khalaji A.D., Feizi N., Kučeráková M., Dušek M. (2015). Synthesis, characterization, crystal structure and antibacterial activity of new sulfur-bridged dinuclear silver(I) thiosemicarbazone complex [Ag2(PPh3)2(μ-S-Brcatsc)2(η1-S-Brcatsc)2](NO_3_)_2_. Inorg. Chim. Acta.

[50] Rageh N.M., Mawgoud A.M.A., Mostafa H.M. (1999). Transition Metal Complexes Derived From 2-hydroxy-4-(p-tolyldiazenyl)benzylidene)-2-(p-tolylamino)acetohydrazide Synthesis, Structural Characterization, and Biological Activities. Chem. Pap..

[51] Lever A.B.P. (1984). Inorganic Electronic Spectroscopy.

[52] Chehelgerdi M., Chehelgerdi M., Allela O.Q.B., Pecho R.D.C., Jayasankar N., Rao D.P., Thamaraikani T., Vasanthan M., Viktor P., Lakshmaiya N. (2023). Progressing nanotechnology to improve targeted cancer treatment: Overcoming hurdles in its clinical implementation. Mol. Cancer.

[53] Prabhakaran P., Hassiotou F., Blancafort P., Filgueira L. (2013). Cisplatin induces differentiation of breast cancer cells. Front. Oncol..

[54] Sethukumar A., Udhaya Kumar C., Agilandeshwari R., Arul Prakasam B. (2013). Synthesis, stereochemical, structural and biological studies of some 2,6-diarylpiperidin-4-one *N*(4′)-cyclohexyl thiosemicarbazones. J. Mol. Struct..

[55] Vijayan P., Vijayapritha S., Ruba C., Viswanathamurthi P., Linert W. (2019). Ruthenium(II) carbonyl complexes containing thiourea ligand: Enhancing the biological assets through biomolecules interaction and enzyme mimetic activities. Monatsh Chem..

[56] Şen B., Kalhan H.K., Demir V., Güler E.E. (2019). Crystal structures, spectroscopic properties of new cobalt(II), nickel(II), zinc(II) and palladium(II) complexes derived from 2-acetyl-5-chloro thiophene thiosemicarbazone: Anticancer evaluation. Mater. Sci. Eng. C.

[57] Piri Z., Moradi–Shoeili Z., Assoud A. (2019). Ultrasonic assisted synthesis, crystallographic, spectroscopic studies and biological activity of three new Zn(II), Co(II) and Ni(II) thiosemicarbazone complexes as precursors for nano-metal oxides. Inorg. Chim. Acta.

[58] Akbari A., Ghateazadeh H., Takjoo R., Sadeghi-Nejad B., Mehrvar M., Mague J.T. (2019). Synthesis & crystal structures of four new biochemical active Ni(II) complexes of thiosemicarbazone and isothiosemicarbazone-based ligands: In vitro antimicrobial study. J. Mol. Struct..

[59] Mayada S.A., Fathy A.E., Mohamad M.E., Sadashiva K., Laila A.J.A. (2023). Synthesis and characterization of thiosemicarbazone metal complexes: Crystal structure, and antiproliferation activity against breast (MCF7) and lung (A549) cancers. J. Mol. Struct..

[60] Ali M.S., Kuraijam D., Karnik S., Jaragh-Alhadad L.A. (2023). Hydrazone bimetallic complex: Synthesis, characterization, in silico and biological evaluation targeting breast and lung cancer cells’ G-quadruplex DNA. Kuwait J. Sci..

[61] Zhu W., Li Y., Gao L. (2015). Cisplatin in combination with programmed cell death protein 5 increases antitumor activity in prostate cancer cells by promoting apoptosis. Mol. Med. Rep..

[62] He Y., Zhu Q., Chen M., Huang Q., Wang W., Li Q., Huang Y., Di W. (2016). The changing 50% inhibitory concentration (IC50) of cisplatin: A pilot study on the artifacts of the MTT assay and the precise measurement of density-dependent chemoresistance in ovarian cancer. Oncotarget.

[63] Ćwiklińska-Jurkowska M., Wiese-Szadkowska M., Janciauskiene S., Paprocka R. (2023). Disparities in Cisplatin-Induced Cytotoxicity—A Meta-Analysis of Selected Cancer Cell Lines. Molecules.

[64] Kotova O., Lyssenko K., Rogachev A., Eliseeva S., Fedyanin I., Lepnev L., Pandey L., Burlov A., Garnovskii A., Vitukhnovsky A. (2011). Low temperature X-ray diffraction analysis, electronic density distribution and photophysical properties of bidentate *N*,*O*-donor salicylaldehyde Schiff bases and zinc complexes in solid state. J. Photochem. Photobiol. A Chem.

[65] Chiş V., Filip S., Miclăuş V., Pîrnău A., Tănăselia C., Almăşan V., Vasilescu M. (2005). Vibrational spectroscopy and theoretical studies on 2,4-dinitrophenylhydrazine. J. Mol. Struct..

[66] Karabacak M., Kose E., Atac A. (2012). Molecular structure (monomeric and dimeric structure) and HOMO–LUMO analysis of 2-aminonicotinic acid: A comparison of calculated spectroscopic properties with FT-IR and UV–Vis. Spectrochim. Acta Part A Mol. Biomol. Spectrosc..

[67] Atac A., Karabacak M., Karaca C., Kose E. (2012). NMR, UV, FT-IR, FT-Raman spectra and molecular structure (monomeric and dimeric structures) investigation of nicotinic acid N-oxide: A combined experimental and theoretical study. Spectrochim. Acta Part A Mol. Biomol. Spectrosc..

[68] Porchelvi E.E., Muthu S. (2015). Vibrational spectra, molecular structure, natural bond orbital, first order hyperpolarizability, thermodynamic analysis and normal coordinate analysis of Salicylaldehyde p-methylphenylthiosemicarbazone by density functional method. Spectrochim. Acta Part A Mol. Biomol. Spectrosc..

[69] Mayada S.A., Mohamad H. (2021). Chelating activity of (2E,2′E)-2,2′-(pyridine-2,6-diylbis(ethan-1-yl-1-ylidene)bis(N-ethylhydrazinecarbothioamide). J. Mol. Struct..

[70] Damena T., Zeleke D., Desalegn T., Demissie T.B., Eswaramoorthy R. (2022). Synthesis, Characterization, and Biological Activities of Novel Vanadium(IV) and Cobalt(II) Complexes. ACS Omega.

[71] Hasan M.M., Ahsan H.M., Saha P., Naime J., Kumar Das A. (2021). Antioxidant, Antibacterial and Electrochemical Activity of (E)-N-(4 (Dimethylamino) Benzylidene)-4H-1,2,4-Triazol-4-Amine Ligand and Its Transition Metal Complexes. Results Chem..

[72] Chebout O., Trifa C., Bouacida S., Boudraa M., Imane H. (2022). Two New Copper (II) Complexes with Sulfanilamide as Ligand: Synthesis, Structural, Thermal Analysis, Electrochemical Studies and Antibacterial Activity. J. Mol. Struct..

[73] Balouiri M., Sadiki M., Ibnsouda S.K. (2015). Methods for invitro evaluating antimicrobial activity: A review. J. Pharm. Anal..

[74] Jaragh-Alhadada L., Behbehania H., Karnik S. (2022). Cancer targeted drug delivery using active low-density lipoprotein nanoparticles encapsulated pyrimidines heterocyclic anticancer agents as microtubule inhibitors. Drug Deliv..

[75] Laila A.J.A., Mayada S.A., Moustafa S.M., Gamaleldin I.H., Fars K.A., Sadashiva K. (2022). Sulfonamide derivatives mediate breast and lung cancer cell line killing through tubulin inhibition. J. Mol. Struct..

[76] Jaragh-Alhadad L., Samir M., Harford T.J., Karnik S. (2022). Low-density lipoprotein encapsulated thiosemicarbazone metal complexes is active targeting vehicle for breast, lung, and prostate cancers. Drug Deliv..

[77] Hembram K.C., Kumar R., Kandha L., Parhi P.K., Kundu C.N., Bindhani B.K. (2018). Therapeutic prospective of plant-induced silver nanoparticles: Application as antimicrobial and anticancer agent. Artif. Cells Nanomed. Biotechnol..

[78] Bisht G., Rayamajhi S. (2016). ZnO Nanoparticles: A Promising Anticancer Agent. Nanobiomedicine.

[79] Arshad F., Naikoo G.A., Hassan I.U., Chava S.R., El-Tanani M., Aljabali A.A., Tambuwala M.M. (2024). Bioinspired and Green Synthesis of Silver Nanoparticles for Medical Applications: A Green Perspective. Appl. Biochem. Biotechnol..

[80] Jaragh-Alhadad L.A., Falahati M. (2022). Tin oxide nanoparticles trigger the formation of amyloid β oligomers/protofibrils and underlying neurotoxicity as a marker of Alzheimer’s diseases. Int. J. Biol. Macromol..

[81] Jaragh-Alhadad L.A., Falahati M. (2022). Copper oxide nanoparticles promote amyloid-β-triggered neurotoxicity through formation of oligomeric species as a prelude to Alzheimer’s diseases. Int. J. Biol. Macromol..

[82] Hoshyar N., Gray S., Han H., Bao G. (2016). The effect of nanoparticle size on in vivo pharmacokinetics and cellular interaction. Nanomedicine.

[83] Ejigah V., Owoseni O., Bataille-Backer P., Ogundipe O.D., Fisusi F.A., Adesina S.K. (2022). Approaches to Improve Macromolecule and Nanoparticle Accumulation in the Tumor Microenvironment by the Enhanced Permeability and Retention Effect. Polymers.

[84] Rümenapp C., Gleich B., Haase A. (2012). Magnetic Nanoparticles in Magnetic Resonance Imaging and Diagnostics. Pharm. Res..

[85] Hoda F.E., Saif M., Mashaly M., Shimaa A., Mohamed F.E., Eid M.F., Nabeel A.I., Fouad R. (2017). New nano-complexes of Zn(II), Cu(II), Ni(II) and Co(II) ions; spectroscopy, thermal, structural analysis, DFT calculations and antimicrobial activity application. J. Mol. Struct..

[86] Sakthivel A., Thangagiri B., Raman N., Joseph J., Guda R., Kasula M., Mitu L. (2021). Spectroscopic, SOD, anticancer, antimicrobial, molecular docking and DNA binding properties of bioactive VO(IV), Cu(II), Zn(II), Co(II), Mn(II) and Ni(II) complexes obtained from 3-(2-hydroxy-3-methoxybenzylidene)pentane-2,4-dione. J. Biomol. Struct. Dyn..

[87] Jain S., Rana M., Sultana R., Mehandi R., Rahisuddin (2022). Schiff Base Metal Complexes as Antimicrobial and Anticancer Agents. Polycycl. Aromat. Compd..

[88] Jan A.T., Azam M., Siddiqui K., Ali A., Choi I., Haq Q.M.R. (2015). Heavy Metals and Human Health: Mechanistic Insight into Toxicity and Counter Defense System of Antioxidants. Int. J. Mol. Sci..

[89] Jaishankar M., Tseten T., Anbalagan N., Mathew B.B., Beeregowda K.N. (2014). Toxicity, mechanism and health effects of some heavy metals. Interdiscip. Toxicol..

[90] Jungwirth U., Kowol C.R., Keppler B.K., Hartinger C.G., Berger W., Heffeter P. (2011). Anticancer activity of metal complexes: Involvement of redox processes. Antioxid. Redox Signal..

[91] Gai S., He L., He M., Zhong X. (2023). Anticancer Activity and Mode of Action of Cu(II), Zn(II), and Mn(II) Complexes with 5-Chloro-2-N-(2-quinolylmethylene)aminophenol. Molecules.

[92] Akl M.A., Mostafa A.G., El-Zeny A.S., El-Gharkawy E.R.H. (2025). Design, spectroscopic analysis, DFT calculations, adsorption evaluation, molecular docking, comprehensive in silico and in vitro bioactivity studies of thiocarbohydrazide grafted dialdehyde cellulose nanobiosorbent. Sci. Rep..

[93] Salehi R., Abyar S., Ramazani F., Khandar A.K., Hosseini-Yazdi S.A., White J.M., Edalati M., Kahroba H., Talebi M. (2022). Enhanced anticancer potency with reduced nephrotoxicity of newly synthesized platin-based complexes compared with cisplatin. Sci. Rep..

[94] Liang W., Huang Y., Wang Y., Lu D., Sun Q. (2025). Research Progress of Platinum-Based Complexes in Lung Cancer Treatment: Mechanisms, Applications, and Challenges. Int. J. Mol. Sci..

[95] Ndagi U., Mhlongo N., Soliman M.E. (2017). Metal complexes in cancer therapy—An update from drug design perspective. Drug Des. Dev. Ther..

[96] Howsaui H.B., Sharfalddin A.A., Abdellattif M.H., Basaleh A.S., Hussien M.A. (2021). Synthesis, Spectroscopic Characterization and Biological Studies of Mn(II), Cu(II), Ni(II), Co(II) and Zn(II) Complexes with New Schiff Base of 2-((Pyrazine-2-ylimino)methyl)phenol. Appl. Sci..

[97] Ngece K., Khwaza V., Paca A.M., Aderibigbe B.A. (2025). The Antimicrobial Efficacy of Copper Complexes: A Review. Antibiotics.

[98] Jaragh-Alhadad L.A., Ali M.S. (2022). Methoxybenzamide derivative of nimesulide from anti-fever to anti-cancer: Chemical characterization and cytotoxicity. Saudi Pharm. J..

[99] Laila A.J.A., Gamaleldin I.H., Fars K.A. (2022). Development of nimesulide analogs as a dual inhibitor targeting tubulin and HSP27 for treatment of female cancers. J. Mol. Struct..

[100] Laila A.J.A., Mayada S.A. (2022). Nimesulide derivatives reduced cell proliferation against breast and ovarian cancer: Synthesis, characterization, biological assessment, and crystal structure. Kuwait J. Sci..

[101] Hugo V.-V.V., Alejandro H.S.M., María V.S.A., María R.H., Antonio L.R.M., Guadalupe P.O.M., Antonio M.G.M., Fernando A.H., Víctor A., Diego C.A. (2018). Molecular Modeling and Synthesis of Ethyl Benzyl Carbamates as Possible Ixodicide Activity. Comput. Chem..

[102] Andersson M.P., Uvdal P. (2005). New Scale Factors for Harmonic Vibrational Frequencies Using the B3LYP Density Functional Method with the Triple-ζ Basis Set 6-311+G(d,p). J. Phys. Chem. A.

[103] Bhat A.R., Dongre R.S., Almalki F.A., Berredjem M., Aissaoui M., Touzani R., Hadda T.B., Akhter M.S. (2021). Synthesis, biological activity and POM/DFT/docking analyses of annulated pyrano[2,3-d]pyrimidine derivatives: Identification of antibacterial and antitumor pharmacophore sites. Bioorg. Chem..

[104] Guerfi M., Berredjem M., Bahadi R., Djouad S.-E., Bouzina A., Aissaoui M. (2021). An efficient synthesis, characterization, DFT study and molecular docking of novel sulfonylcycloureas. J. Mol. Struct..

[105] Shakdofa M.M.E., Morsy N.A., Rasras A.J., Al-Hakimi A.N., Shakdofa A.M.E. (2020). Synthesis, characterization, and density functional theory studies of hydrazone–oxime ligand derived from 2,4,6-trichlorophenyl hydrazine and its metal complexes searching for new antimicrobial drugs. Organomet. Chem..

[106] Mohan B., Choudhary M. (2021). Synthesis, crystal structure, computational study and anti-virus effect of mixed ligand copper (II) complex with ONS donor Schiff base and 1, 10-phenanthroline. J. Mol. Struct..

[107] Andrew F.P., Ajibade P.A. (2018). Synthesis, characterization and anticancer studies of bis(1-phenylpiperazine dithiocarbamato) Cu(II), Zn(II) and Pt(II) complexes: Crystal structures of 1-phenylpiperazine dithiocarbamato-S,S′ zinc(II) and Pt(II). J. Mol. Struct..

[108] Agnieszka D.K., Liliana M. (2011). Structural studies and characterization of 3-formylchromone and products of its reactions with chosen primary aromatic amines. J. Mol. Struct..

[109] Saif M., Hoda F.E., Mahmoud M., Eid M.F., Nabeel A.I., Fouad R. (2018). Hydrothermal preparation and physicochemical studies of new copper nano-complexes for antitumor application. J. Mol. Struct..

[110] Damena T., Alem M.B., Zeleke D., Desalegn T., Eswaramoorthy R., Demissie T.B. (2022). Novel Zinc(II) and Copper(II) Complexes of 2-((2-Hydroxyethyl)amino)quinoline-3-carbaldehyde for Antibacterial and Antioxidant Activities: A Combined Experimental, DFT, and Docking Studies. ACS Omega.

